# CRISPR/Cas9 generated DSB clusters mimic complex lesions induced by high-LET radiation and shift repair from c-NHEJ to mutagenic repair pathways

**DOI:** 10.1038/s41598-025-22945-9

**Published:** 2025-10-20

**Authors:** Emil Mladenov, Mathias Kallies, Martin Stuschke, Eleni Gkika, George Iliakis

**Affiliations:** 1 Division of Experimental Radiation Biology, Department of Radiation Therapy, University Hospital Essen, University of Duisburg-Essen, Hufelandstr. 55, 45147 Essen, Germany; 2 Institute of Medical Radiation Biology, University Hospital Essen, University of Duisburg-Essen, 45147 Essen, Germany; 3 German Cancer Consortium (DKTK), Partner Site University Hospital Essen, German Cancer Research Center (DKFZ), 45147 Essen, Germany; 4 Radiation Biology Laboratory, Department of Radiotherapy and Radiation Oncology, University Hospital Bonn, 53127 Bonn, Germany

**Keywords:** DSB complexity, DSB clusters, DNA DSB repair, CRISPR/Cas9, high-LET radiation, *HPRT* gene, c-NHEJ, Homologous recombination, alt-EJ, DNA damage and repair, Mutation

## Abstract

DNA double-strand break (DSB) clusters are a hallmark of high-linear energy transfer (high-LET) radiation and are associated with pronounced biological effects, including reduced cell survival and elevated genomic instability. Our previous work in Chinese hamster cells, engineered with variably designed clusters of I-SceI recognition sites, integrated at multiple genomic locations, revealed that DSB clusters suppress classical non-homologous end-joining (c-NHEJ) and induce chromosomal translocations that ultimately increase cell lethality. Here, we extend this line of investigation to human cell lines and generate DSB clusters using alternative approaches that do not require prior genetic manipulation of the test cell lines. We employ CRISPR/Cas9-technology to generate DSB clusters of specific design at a selected genomic locus and examine their consequences on locus integrity. We target Exon 3 of the human HPRT (*hHPRT*) gene and introduce single DSBs or DSB clusters of varying numbers and inter-DSB distances. Alterations at the locus reflecting *hHPRT* gene inactivation, are quantified as mutations causing resistance to 6-thioguanine (6TG). Our results show that DSB clusters are markedly more potent inducers of mutations than single DSBs and that DSBs spaced within ~ 600 base pairs synergize in mutation induction. Mechanistic analyses using small-molecule inhibitors and engineered gene knockout cell lines reveal that the increased mutagenicity of clustered DSBs is primarily mediated by DNA end resection and PARP1-dependent alternative end-joining (alt-EJ) pathways. These findings reinforce the biological relevance of DSB clusters as a severe form of complex DNA damage and provide mechanistic insights into high-LET radiation-induced increased cell killing and genomic instability.

## Introduction

DNA double-strand breaks (DSBs) are widely recognized as highly cytotoxic lesions with carcinogenic potential. In the course of evolution, mammalian cells have evolved four distinct mechanisms to repair DSBs and mitigate their harmful biological consequences. Homologous recombination (HR) is the only error-free repair pathway, capable of restoring both DNA integrity and sequence at the break site. However, HR is restricted to the post-replication stages of the cell cycle, where the required sister chromatids are available. Classical non-homologous end-joining (c-NHEJ) operates throughout the cell cycle and provides means for the rapid removal of DSBs from the genome^1–3^. However, c-NHEJ is prone to errors, as there are no build-in mechanisms to ensure restoration of DNA sequence at the junction, or the rejoining of the original DNA ends^4,5^. Alternative end-joining (alt-EJ), also referred to as microhomology mediated end-joining or backup end-joining, is believed to be hyper-activated when HR or c-NHEJ are unavailable or fail to engage properly in the repair^5–9^. Compared to c-NHEJ, alt-EJ operates with slower kinetics, lower efficiency and greater proneness for errors, often manifesting as translocations or deletions in the genome^5,10^. Single-strand annealing (SSA) is also an error-prone repair mechanism owing to the generation of large deletions, between the repetitive DNA segments utilized in repair^11^.

HR, alt-EJ and SSA are categorized as DNA end resection-dependent DSB repair pathways, as they share this critical processing step^12^. During DNA end resection, the MRN complex, together with CtIP, initiates short-range DNA degradation of the 5’-strand, which is subsequently extended by the coordinated action of BLM/DNA2 complex, or by the EXO1 exonuclease^10,13^. The resulting single-stranded DNA (ssDNA) is essential for the initiation of HR and SSA and also facilitates alt-EJ by exposing microhomologies^14^. POLθ plays a key role in alt-EJ, by mediating the annealing of the resected 3′-overhangs and extending one of the DNA 3′-OH ends using the annealed partner as a template^15,16^. Alt-EJ efficiency is also promoted by PARP1, but it is not clear whether PARP1-dependent alt-EJ requires extensive resection^17–21^.

Over the past decades, several pieces of evidence suggested that ionizing radiation (IR)-induced DSBs comprises forms with different biological severity^22–25^. Notably, high linear energy transfer (high-LET) radiation induces DSBs with increased toxicity, presumably owing to the increased ionization clustering along the particle track, which in-turn generates DNA lesions of elevated molecular complexity.

Traditionally, complexity is defined by the presence of DNA modifications in the vicinity of the DSB^26^. However, defined biological models of such classically outlined complex DSBs are not yet available, and the known biological responses of such lesions do not fully explain well-characterized effects of high-LET radiation. In addition, studies following the accretion of DSB repair proteins at the break sites, suggest that after exposure to high-LET IR, clusters of repair proteins are spaced at distances between 50 and 100 nm, that could expand up to 1.5 μm, making the underpinning “complex” lesions significantly larger than the 10 bp invoked in the classical definition^27–29^.

To address this gap, our laboratory explores, without discounting the classical definition, alternative definitions of DSB complexity, with a strong focus on DSB clusters. The relevance of DSB clusters in the cytotoxic effects of different radiation modalities is supported by extensive computational modeling^30–32^. Notably, the hypothesis of DSB cluster relevance in radiation responses can be experimentally tested in molecularly defined model systems^24,33^. Indeed, we showed in Chinese hamster ovary (CHO) cells that compared to single-DSBs, clusters of DSBs generated by the I-SceI meganuclease at multiple, appropriately engineered genomic sites, destabilize chromatin and compromise c-NHEJ similarly to high-LET IR. DSB clusters also markedly increase cell killing and translocation formation^24,33,34^. Translocation formation by DSB clusters utilizes PARP1 activity, implicating alt-EJ in their formation. Furthermore, immunofluorescence experiments show that single-DSBs and DSB clusters similarly provoke the formation of distinct γH2AX foci, suggesting activation of early DNA damage response (DDR), similar to single-DSBs. Live-cell imaging also shows similar single-focus recruitment of the early-response protein MDC1, both to single-DSBs and DSB clusters, while the late-response protein, 53BP1, shows stronger recruitment to DSB clusters^33^.

Here, we extend our previous work in CHO cells to human cell lines and assess the mutagenic potential of DSB clusters by leveraging CRISPR/Cas9 technology. We introduce DSB clusters of varying numbers and inter-lesion distances within Exon 3 of the human HPRT (*hHPRT*) gene and quantify mutation induction via 6-thioguanine resistance (6TG^r^). This approach has been extensively used in the past to study IR-induced mutagenesis and extensive work is available after exposure to both low- and high-LET radiation modalities^35–40^. In the present study, we introduce individual DSBs or DSB clusters of two, three and four DSBs and evaluate the role of DSB repair pathways in their processing with emphasis on misrepair events leading to the generation of mutant cells in this model system. Effects on cell survival of such constellations of DSBs, directed to a single genomic locus, are not part of the present study.

## Materials and methods

### Cultivation and propagation of cell lines

A549 cells were isolated from the lung tissue of a 58-year-old Caucasian male with lung cancer and obtained from the ATCC. The A549 derivative, A549-copGFP-Cas9 cell line was purchased from GeneCopoeia (SL504). This cell line stably expresses the Cas9 nuclease, integrated into the human AAVS1 “safe harbor” locus (also known as PPP1R2C), together with copGFP protein and a hygromycin resistance cassette. Parental A549 and A549-Cas9-copGFP cells, as well as A549-*PRKDC*^*−/−*^, A549-*ATM*^*−/−*^ and A549-*PARP1*^*−/−*^ cells, generated by CRISPR/Cas9 mediated knock-out of the corresponding gene (for details see below), were grown in McCoy’s 5 A medium, supplemented with 10% fetal bovine serum (FBS) and Pen/Strep antibiotic mixture. All cell lines were grown at 37 °C in a humidified incubator with 5% CO_2_. In order to keep cells in the exponential phase of growth, cells were passaged every two days using 100 mm cell culture dishes following standard cell culture handling procedures.

### Treatment with DNA repair inhibitors

A specific DNA-PKcs inhibitor, 8-(4-Dibenzothienyl)-2-(4-morpholinyl)-4 H-1-benzopyran-4-one (NU7441, DNA-PKcsi) with IC_50_ for DNA-PKcs of 13 nM was dissolved in DMSO at 10 mM stock concentration and was used at 5 µM final concentration (Tocris Bioscience). A specific ATM inhibitor, 2-(4-Morpholinyl)-6-(1-thianthrenyl)-4 H-pyran-4-one (KU55933, ATMi) with IC_50_ for ATM of 2.2 nM, was used at 10 µM final concentration from a DMSO stock solution (10 mM) (Tocris Bioscience). The PARP1 inhibitor PJ34 (PARP1i) was dissolved in DMSO at 10 mM concentration and was used at 10 µM. L67, a LIG1/LIG3 inhibitor (LIG1/3i), was dissolved in DMSO and was used at 100 µM final concentration. All inhibitors were administrated to the cells 4 h after transfection with a selected combination of Cas9/gRNA expression vectors, and were kept in the growth media during the mutation expression phase that was determined to be 2–4 days.

### Transfection of cells with Cas9 and gRNA expression vectors

Exponentially growing cells were used in all transfection procedures. hCas9wt (Addgene#41815)^41^, hCas9D10A (Addgene#41816)^41^, Cas9-GFP (Addgene#44719)^42^ and gRNAs expression vectors^41^ were delivered to the cells by nucleofection using the Amaxa 2D nucleofector device (Lonza), by applying the standard A549 transfection protocol, provided by the manufacturer. The total amount of plasmid used for transfection reaction was 1 µg per 1 × 10^6^ cells. A total of 3 × 10^6^ cells were transfected per sample. After transfection, cells were plated in 3 × 100 mm dishes and cells were incubated for 2–4 days, before plating for the forward mutation assay.

### Generation of knock-down cells after transfection with specific SiRNAs

Exponentially growing A549 or A549-Cas9-copGFP cells were transfected by nucleofection with the corresponding siRNA, targeting the DNA-PKcs or CtIP. Briefly, about 3–5 × 10^6^ cells were transfected with Amaxa 2D nucleofector device by applying the standard A549 transfection protocol, provided by the manufacturer. The following siRNA sequences were used: DNA-PKcs (CGGCUAACUCGCCAGUUUA) and CtIP (GCUAAAACAGGAACGAAUC). The confirmation of the successful knock-down was monitored by western blot (WB) analysis of the corresponding proteins.

### Generation of DSB repair deficient A549 cell lines

We used CRISPR/Cas9 technology to generate knockout cell lines in parental A549 cells. To achieve this, specific gRNAs targeting the *PRKDC*, *ATM* or *PARP1* genes were designed and cloned into gRNA expression/cloning vector^41^. For each gene, two gRNAs were selected. The parental A549 cells were transfected with the constructed gRNA plasmid vectors containing the selected gRNAs and were co-transfected with a plasmid expressing GFP-tagged Cas9 endonuclease, Cas9-GFP (Addgene# 44719)^42^. The target sequences were as follow:

#### DNA-PKcs

gRNA1: AAAGGCATCAACTCAGGGAC, located at Chr 8, *PRKDC*, ENSG00000253729 (position 47777846–47777865);

gRNA2: CAGCAAGTGCACCTGTGTAG, located at Chr. 8, *PRKDC*, ENSG00000253729 (position 47778447–47778466).

#### ATM

gRNA1: ATCATTAAGTACTACACTCA, located at Chr. 11, *ATM* exon 2;

gRNA2: CTCTATCATGTTCTAGTTGA), located at Chr. 11, *ATM* exon 2.

#### PARP1

gRNA1: ACTAGAAGGAAGAGAAACAG, located at Chr. 1, *PARP1* exon 1;

gRNA2: GGCCCGCACCTGCACCATGA, located at Chr. 1, *PARP1* exon 1.

Following transfection, cells were allowed to recover for 3–4 days and were subsequently sorted based on GFP expression (MoFlo Astrios, Beckman Coulter). The sorted cells were cultured for 10 days to allow for colony formation. The resulting colonies were expanded to a population of approximately 3–4 × 10⁶ cells, which were either cryopreserved or subjected to further analysis via western blotting or indirect immunofluorescence to evaluate protein levels and activity of DNA-PKcs, ATM and PARP1. Clones exhibiting efficient knockdown were selected for further study.

### Design and cloning of CRISPR/Cas9 guide RNAs (gRNAs) that target the hHPRT locus

**Fig. 1:**
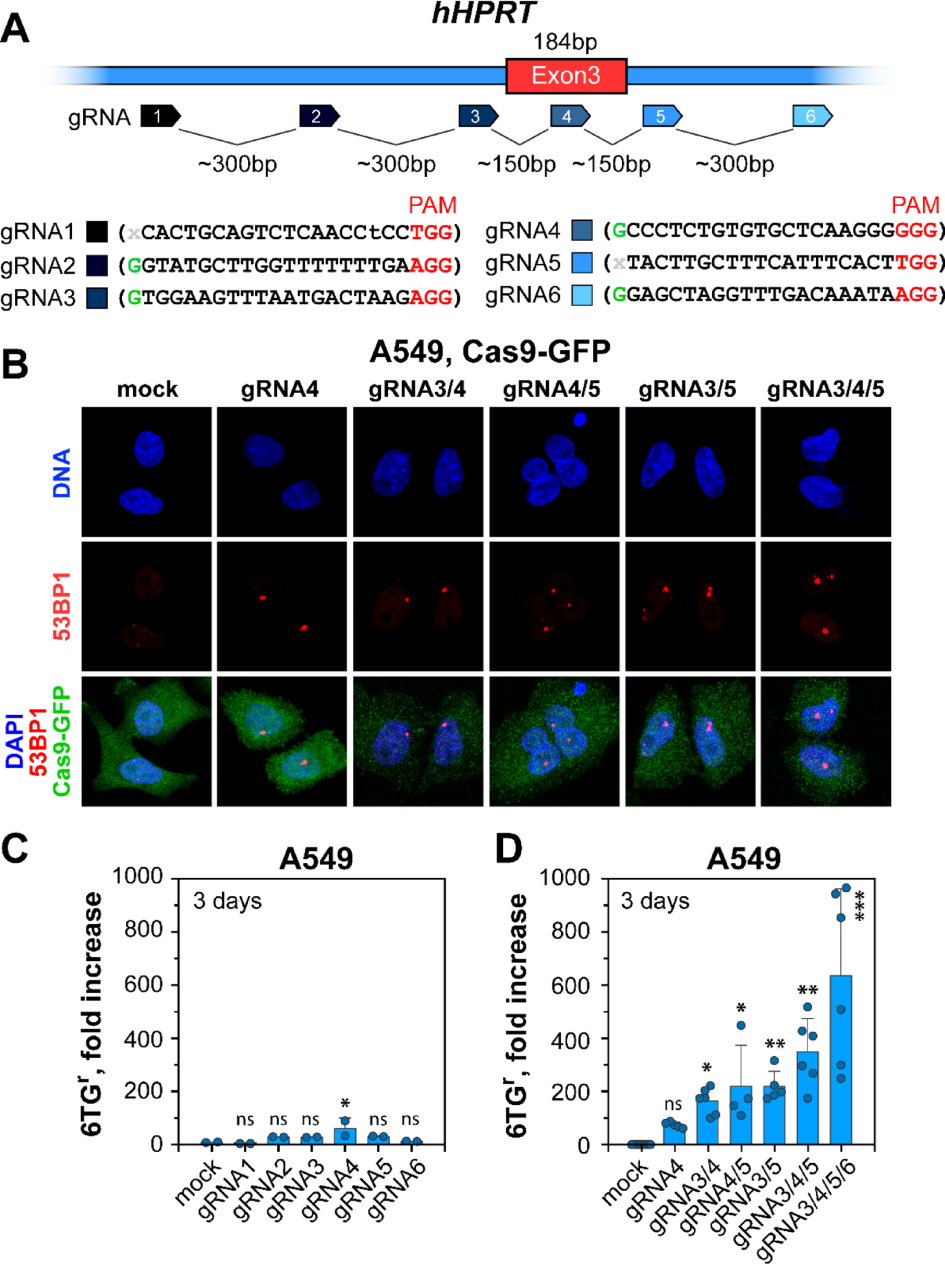
*CRISPR/Cas9 efficiently generates single DSBs and DSB clusters at the hHPRT locus*. (**A**) Schematic representation of Exon 3 of the *hHPRT* gene showing the target sites of the guide RNAs (gRNAs) utilized. The distances between DSBs are indicated in base pairs (bp). The nucleotide sequences of the gRNAs are provided in the lower part. The flanking 5’-nucleotides, as well as the corresponding PAM sequences are indicated. (**B**) Representative images of 53BP1 foci after co-transfection of A549 cells with Cas9-GFP and the indicated combinations of gRNA expression vectors. Only GFP positive cells are monitored in these experiments. (**C**) Fold increase in mutation frequency (MF) in A549 cells transfected with gRNA expression vectors inducing single DSBs at the indicated locations in and around Exon 3 of *hHPRT*. Data represents means and standard deviations (SD) from two biological repeats. Individual data points from each determination are also shown. (**D**) Fold increase of MF in A549 cells, 3 days after transfection with combinations of gRNA expression vectors inducing DSB clusters of increasing complexity, i.e. increasing number of DSBs. The background MF for the selected experiments was between 1.05 × 10^− 5^ and 1.86 × 10^− 5^ (Table S2). The bar plot represents the mean and standard deviation (SD) from 4 to 6 independent experiments. Dots indicate the individual values determined in each experiment. The corresponding P-values are shown in Table S1.

The gRNAs for the *hHPRT* locus were designed to target Exon 3 and its flanking sequences in order to generate two, three or four DSBs at varying distances. The exact location of the target sequences is shown in Fig. 1A. The guide RNA (gRNAs) sequences were as follows:

- gRNA1 ( CACTGCAGTCTCAACCTCC ), located at Chr. X, *hHPRT* intron 3,
- gRNA2 ( GGTATGCTTGGTTTTTTTGA ), located at Chr. X, *hHPRT* intron 3.
- gRNA3 ( GTGGAAGTTTAATGACTAAG ), located at Chr. X, *hHPRT* intron 3.
- gRNA4 ( GCCCTCTGTGTGCTCAAGGG ), located at Chr. X, *hHPRT* exon 3.
- gRNA5 ( TACTTGCTTTCATTTCACT ), located at Chr. X, *hHPRT* intron 4.
- gRNA6 ( GGAGCTAGGTTTGACAAATA ), located at Chr. X, *hHPRT* intron 4.

The corresponding gRNAs were cloned into the gRNA expression vector developed by Church’s lab^41,43^ and obtained from Addgene (Addgene#41824), using Gibson assembly according to the authors’ instructions^41^.

### hHPRT-forward-mutation-assay

For the *hHPRT* forward mutation assay, parental A549 or A549 cells deficient for DNA-PKcs, ATM or PARP1, as well as A549-Cas9-copGFP cells, were transfected with a mixture of Cas9 and gRNA expression vectors at equimolar concentrations. After transfection, cells were incubated under normal growth conditions for 2, 3 or 4 days to manifest the mutation at the *hHPRT* locus as loss of HPRT function. Subsequently, cells were trypsinized, counted and adjusted to 0.4 × 10^6^ cells/mL. Approximately 0.1 × 10^6^ cells were plated in triplicates in 60 mm dishes containing 5 ml of growth medium supplemented with 6TG (5 µg/mL). For mock transfected controls the number of plated cells was 3–5 times higher. In addition, 300 cells from each condition were plated in 6TG-free media to determine the plating efficiency (PE). All dishes were plated in triplicates and were incubated at 37 °C in a humidified atmosphere of 5% CO_2_ in air for 10 days. After this period of colony growth, the culture medium was removed and cells were fixed and stained for 5 min in 1% crystal violet, dissolved in 70% ethanol. Colonies at all conditions were either manually counted, or assessed using a colony counting software (Cell counter, by Nghia Ho, ver. 2021.1.17).

The mutation frequency (MF) was calculated by counting the number of colonies obtained after 6TG selection (Nc), divided to the number of plated cells (Np), corrected for the plating efficiency (PE) of cells plated in non-selective media. These MF values were further corrected for transfection efficiency (TE, %) that was determined in each experiment.

$${\text{MF }}={\text{ }}\left( {{\text{Nc}}/{\text{Np}}} \right){\text{ x }}\left( {{\text{1}}/{\text{PE}}} \right){\text{ x }}\left( {{\text{1}}00/{\text{TE}}} \right)$$

$${\text{PE}}\,=\,{\text{number of cells plated}}/{\text{number of cells counted}}$$

### Indirect Immunofluorescence

Immunofluorescence (IF) experiments were carried out for the validation of DSB formation, after transfection with gRNAs and Cas9 expression vectors. For testing the efficiency of each gRNA, cells were seeded at a concentration of 0.2 × 10^6^ cells per dish immediately after transfection and coverslips were processed 2 days later. For validation of PARP1 and ATM knockout, cells were seeded at a concentration of 0.1 × 10^6^ for 2 days and were either exposed to 1 Gy x-rays or treated for 15 min with 10 mM with H_2_O_2_. Following damage induction, cells were washed once with PBS and were fixed in 2 ml fixation buffer (3% PFA, 2% sucrose in 1 × PBS) for 15 min at room temperature (RT). After fixation, cells were permeabilized in 2 ml of P-solution (100 mM Tris pH 7.4, 50 mM EDTA, 0.5% Triton X-100) for 7 min at RT. Cells were washed once with PBS and were incubated in 2 ml of PBG-blocking buffer (0.2% gelatin, 0.5% BSA fraction V in 1× PBS) overnight at 4 °C. Cells were incubated at RT for 1.5 h with the following primary antibodies: mouse-monoclonal, anti-53BP1 (53BP1(E10), Santa Cruz Biotechnology, sc-515841), mouse-monoclonal, anti-pATM-S1981 (Phospho-ATM (Ser1981) (10H11.E12), Cell Signaling, #4526), mouse-monoclonal, anti-γH2AX (Histone H2A.XS139ph [GT2311], GeneTex, GTX628789), and mouse-monoclonal, anti-PAR (pADPr Antibody (10 H), Santa Cruz Biotechnology, sc-56198) - all diluted to the company recommended dilutions in PBG. Coverslips were washed three times with PBS and were incubated for 1.5 h with the corresponding secondary antibody conjugated with Alexa Fluor 488 (Alexa Fluor^®^ 488 Goat anti-Mouse IgG (H + L), Thermo Fisher scientific, A-11001), or Alexa Fluor 568 (Alexa Fluor^®^ 568 Goat anti-Mouse IgG (H + L), Thermo Fisher scientific, A-11004), or Alexa Fluor 647 (Alexa Fluor^®^ 647 Goat anti-Mouse IgG (H + L), Thermo Fisher scientific, A-21235), all diluted 1/400 in PBG-blocking buffer. DNA in cells was counterstained with DAPI (1 µg/ml) for 10 min at RT. Finally, cells were washed with PBS and coverslips were mounted on microscope slides using PromoFluor Antifade Reagent. Slides were scanned using Leica SP5 confocal microscope or ZEISS AxioScan.Z1 slide scanner, and images were processed by using the Imaris 9.5.1 (Bitplane) or ZEISS ZEN 3.11 (Carl Zeiss) software.

### Western blot analysis

Following protein extraction in RIPA buffer (Thermo Fisher Scientific), according to the manufacturer’s instructions, the Bradford assay was used to determine the total protein concentration. For most of the WB analyses, 50 µg of total protein extract was mixed with Laemmli loading buffer and samples were loaded on SDS-PAGE. Proteins were resolved on 4–20% gels, until the loading buffer dye exited the gel. WB samples resolved on SDS-PAGE were transferred onto nitrocellulose membranes that were blocked after the transfer with Intercept Blocking Buffer (IBB) (Li-COR Biosciences). Primary antibodies were dissolved in IBB and the membranes were incubated overnight at 4 °C under constant shaking. The following primary antibodies were used: anti-DNA-PKcs (DNA-PK_CS_ antibody (G-4), Santa Cruz Biotechnology, sc-5282), anti-ATM (ATM (D2E2) Rabbit mAb, Cell Signaling, #2873), anti-CtIP (CtIP (D76F7) Rabbit mAb, Cell Signaling, #9201), anti-PARP1 (Anti-PARP antibody, Sigma-Aldrich, P7605), anti-KU80 (Ku80 antibody [N3C2], GeneTex, GTX109935), anti-pKAP1-S824 (Phospho KAP-1 (S824) Antibody, Bethyl Laboratories, A300-767 A), anti-KAP1 (Anti-KAP1 antibody [20C1], Abcam, ab22553), anti-GAPDH (Anti-GAPDH (GA1R) Loading Control, UBPBio, Y1041), and anti β-Actin (beta Actin antibody, GeneTex, GTX109639). After primary antibody incubation, the membranes were washed three times for 10 min each with TBS-T (25 mM Tris-HCl, pH 7.6, 150 mM NaCl, and 0,05% Tween-20) and were incubated for 1.5 h at RT, with appropriate secondary antibodies, conjugated with IRDye680 or IRDye800 (Li-COR Biosciences, 926-68021, 926-68020, 926-32211, 926-32210), diluted 1/10,000 in IBB. After incubation, membranes were washed 3 times with TBS-T and were dried for scanning on Odyssey^®^ M Infrared Imaging System (Li-COR Biosciences). Uncropped WB membranes for all western blot results are summarized and shown as supplementary information in Figures S4, S5 and S6.

### Surveyor nuclease assay

For the Surveyor nuclease assay, A549 cells were co-transfected with Cas9 and single gRNAs vectors, as indicated in the previous section. Three days after transfection cells were collected and genomic DNA (gDNA) was isolated. The gDNA was used as a template in PCR reactions with primers flanking the gRNAs cutting side by using the EnGen ^®^ Mutation Detection Kit (New England Biolabs, NEB#E3321), following the manufacturer protocol. Primer pairs for gRNA1, gRNA2, combined gRNA3/4/5 and gRNA6 were used (Figure S1A). The recommended amount of the obtained PCR products (gRNA1 ~ 480 bp, gRNA2 ~ 310 bp, gRNA3/4/5 ~ 750 bp and gRNA6 ~ 210 bp) were reannealed and were used as substrates for T7-Endonuclease I (T7E1), according to the manufacturer’s instructions. The processed DNA products were run on 2% agarose gels and visualized by SYBR™ Gold staining on Li-COR Odyssey^®^ M Imaging System (Li-COR Biosciences).

### Statistical analysis

For statistical analysis the One-Way ANOVA with Tukey or Fisher post-hock test, implemented in the Origin Pro 2023 software, was applied to the corresponding data sets. For comparative statistical evaluation, the Pair-sample t-Test statistical analysis was carried out using the Origin Pro 2023 software. The following annotations were used to indicate the significance level: ns (*P* > 0.05), * (*P* ≤ 0.05), ** (*P* ≤ 0.01), *** (*P* ≤ 0.001). The calculated significance values for all relevant experiments are shown as supplementary information in Table S1.

## Results

### Harnessing CRISPR/Cas9 technology to generate single DSBs and DSB clusters at the hHPRT locus

The ability of CRISPR/Cas9 to generate DSBs at specific locations in the cellular genome has revolutionized genetic engineering and gene therapy and is presently considered in a wide spectrum of technologies^41,44–46^. Here, we harness this technology to generate single DSBs or DSB clusters of specific designs, to compare their potential to induce mutations, broadly defined here as locus disruption or gene inactivation. We selected the *hHPRT* locus on the X chromosome, because it has been extensively used in previous research, and a wealth of information is available^47–49^. HPRT inactivation, primarily by deletions^35–38,40^, leads to 6TG resistance that can be conveniently quantitated.

The *hHPRT* locus comprises nine exons spanning ~ 50 kilobases (kb)^50^. Exon 3, with a size of 184 base pairs (bp), is the largest and a known mutational hotspot^51^. We designed gRNAs targeting Exon 3 directly (gRNA4), as well as its 5’- and 3’-flanking regions (gRNA1, gRNA2, gRNA3, gRNA5, and gRNA6) (Fig. 1A). By mixing gRNAs in the transfection reaction, we generated either single DSBs or DSB clusters at selected locations and distances from each other (Fig. 1A) to study mutation frequency (MF) quantitated as 6TG resistance (6TG^r^).

To evaluate the “cutting” efficiency of gRNAs, we applied the T7E1 nuclease assay and analyzed restriction products, indicating heteroduplex DNA formation during the Cas9-mediated DSB processing (Figures S1A, S1B, and S1C). For all gRNAs tested, we were able to detect restriction products that reflect the formation of DSBs. The quantification analysis of the restricted products revealed similar “cutting” efficiency for gRNA1, gRNA2, gRNA3, and gRNA5 (Figure S1C). The highest “cutting” efficiency was observed for gRNA4, while the lowest for gRNA6 (Figure S1C). However, the quantitative similarities of the restriction products observed in cells transfected with Cas9 and single gRNA expression vectors suggests that the “cutting” efficiency of the selected gRNA is comparable for all tested conditions.

To confirm that different gRNAs cause DSBs in the genome at similar efficiency, we also scored 53BP1 (Figs. 1B, S1D, and S1E) and γH2AX foci (Figure S1F) in cells, 48 h after transfection with the appropriate expression vectors. We used here a vector expressing a chimeric Cas9 protein fused to GFP (Cas9-GFP) together with plasmids encoding selected gRNAs and analyzed foci formation in GFP positive cells. Representative IF images shown in Figs. 1B, S1D, and S1E indicate the formation of mostly single, large 53BP1 foci in the majority of GFP-positive cells. However, large 53BP1 foci were absent in cells transfected only with the Cas9-GFP expression vector. γH2AX foci were also observed in Cas9-GFP/gRNAs transfected cells, but not in cells transfected with the Cas9-GFP expressing vector alone (Figure S1F). We conclude that our experimental conditions ensure efficient induction of DSBs in transfected cells with all gRNAs tested. Furthermore, we monitored the transfection efficiency in each experiment by quantitating GFP-positive cells, which was in the range between 75 and 88%, and was used to correct mutation induction (Figure S2A).

### DSB-clusters are much more efficient in inducing HPRT mutations than single DSBs

In preliminary experiments, we determined the concentration of 6TG that is required to kill A549 cells carrying a wild-type *HPRT* gene. Figure S2B shows that 6TG concentrations above 1.5 µg/mL reduce significantly colony formation, while above 5 µg/mL no colonies develop. Therefore, 5 µg/mL 6TG was chosen for the selection of mutations in all subsequent experiments. We also examined post-transfection incubation time required for “expression” of the mutation, which includes DSB induction and the degradation or dilution of existing HPRT protein in the generated mutants. Figure S2C shows that 2–4 days of growth are sufficient for the manifestation of mutations and are therefore interchangeably used in subsequent experiments.

Figure 1C and D demonstrate that a single DSB generated by gRNA4 increases mutation frequency by ~ 55-folds - from ~ 1.7 ×  10^–5^(background) to 94.9 × 10^–5^(Figure S2D). Mutations were also induced by the other gRNAs tested (Fig. 1C), but again gRNA4 was clearly the most efficient, possibly owing to the fact that it is the only one that directly targets Exon 3 (Fig. 1A) and show the highest “cutting” efficiency (Figure S1C). Indeed, such DSBs may be repaired with small errors causing mutations, while small errors outside the gene are less likely to compromise HPRT activity.

Strikingly, introducing clusters comprising two DSBs, increased mutation induction ~ 200-fold above the background level (Fig. 1D). Increasing cluster complexity to 3 DSBs further elevated MF to ~ 532 × 10⁻⁵ and clusters of 4 closely spaced DSBs resulted in mutation induction of up to 681 × 10^–5^, or approximately 600-fold above the background (Fig. 1D and S2D). These results demonstrate that DSB clusters are markedly more efficient mutation inducers than single DSBs and that their efficacy increases with increasing number of DSBs per cluster. In line with our previous work^24,33,34,52^, we propose that this increase derives from chromatin destabilization generated by DSB clusters that shifts DSB repair to error-prone processing.

### hHPRT mutations originate from DSBs

**Fig. 2:**
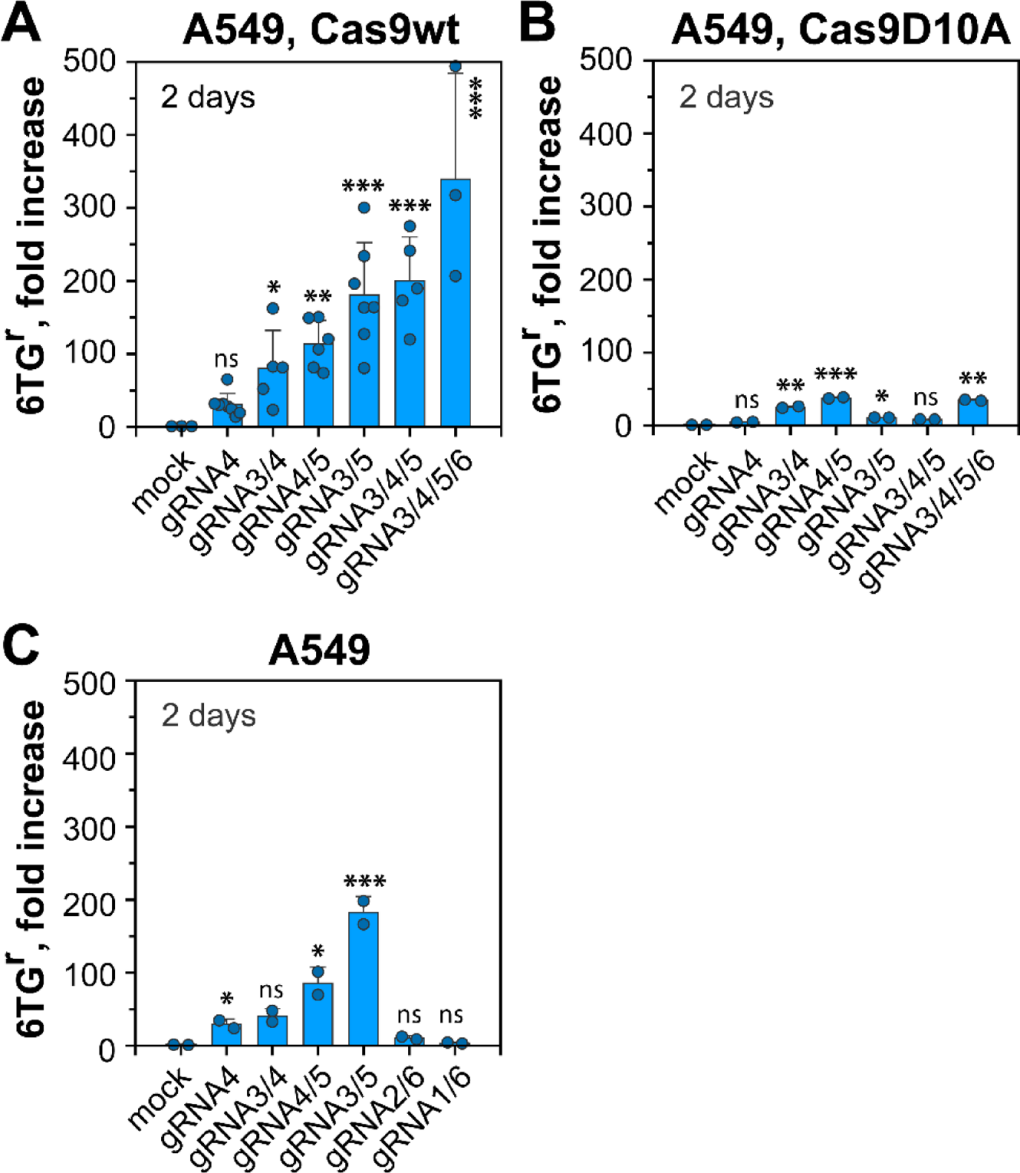
*DSB clusters increase mutation frequency at Exon 3 of hHPRT.* (**A**) Fold increase in MF in A549 cells transfected with wild-type Cas9 (Cas9wt) and the corresponding gRNA expression vectors, plated 2 days after transfection in 6TG-selective growth media. The background MF for the selected experiments was between 0.25 × 10^− 5^ and 1.31 × 10^− 5^ (Table S2). The bar plot represents the mean and SD from 3 to 7 individual experiments. Dots reflect the values of MF determined in each experiment. (**B**) Fold increase in MF of A549 cells transfected with Cas9D10A (“Cas9-nickase”), that generates single-strand breaks (“nicks”). Data is from two biological repeats and the dots show the raw values of each repeat. (**C**) Analysis of MF in A549 cells transfected with the indicated combinations of gRNA expression vectors, and plated 2 days after transfection in 6TG-selective media. The data represents the mean and SD from two experiments. Dots represent the raw values of each repeat. The calculated P-values for all panels are shown in Table S1.

To exclude secondary effects of Cas9 on mutation induction, we compared wild-type Cas9 to “Cas9-nickase” mutant (Cas9D10A). This mutant only induces a single-strand break (SSB) at the target site^53^. If DSBs or DSB clusters are essential for the observed increases in mutation induction, this variant should produce mutants at reduced rates. Indeed, while DSB-dependent inactivation of *hHPRT* gene is highly elevated 2 days after DSB induction (Fig. 2A), HPRT mutations were practically undetectable when SSB are induced (Fig. 2B). Strongly reduced mutation frequency was also apparent when combining gRNAs to generate SSB clusters as compared to DSB clusters (Fig. 2A and B). However, low levels of HPRT mutants were generated from SSB clusters, likely owing to destabilizing interactions causing secondary DSBs. We conclude that the generation of DSBs at the *hHPRT* locus is a key requirement for gene inactivation and mutation induction.

To further investigate the effect of DSB clustering in gene inactivation and mutation induction, we extended the panel of gRNAs to cover a 1200 bp region of the *hHPRT* locus by including two additional gRNAs (gRNA1 and gRNA2) as shown in Fig. 1A. It is evident that generating two DSBs at a distance of ~ 150 bp (gRNA3/4 and gRNA4/5), caused a marked but equivalent increase in MF (Fig. 2C). Notably, when combining gRNA3/5 that closely flank Exon 3, we observed a further increase in MF. This increase likely reflects the loss of Exon 3 as a consequence of the flanking DSBs. In contrast, cells transfected with plasmids expressing gRNAs generating DSBs distant from Exon 3 (e.g., ~ 1200 bp apart, gRNA1/6, or ~ 900 bp apart, gRNA2/6), develop mutations at levels lower than those of single DSBs (Fig. 2C). We conclude that at these distances, DSBs can be processed independently and fail to destabilize chromatin and cause deletions underpinning mutations. Closely located DSBs may also compromise c-NHEJ^24,27,54^. Evidence for such effect is provided next by analyzing HPRT mutations in DSB repair deficient cells.

### DSB clusters suppress c-NHEJ and promote mutagenic DSB repair pathways

The work summarized above shows that while single DSBs in the *hHPRT* locus cause mutations, DSB clusters are much more efficient in this regard. Also, since in a deletion mutant, the integrity of the X chromosome is restored, mutation represents a misrepair event, which raises the question as to the repair pathways involved^55–57^. Among DSB repair pathways, only HR is error-free, while c-NHEJ, alt-EJ and SSA remove DSBs but with errors, including large deletions, particularly when alt-EJ or SSA are involved. In addition, some forms of DSBs functionally compromise the enzymatic machinery of a particular repair pathway, as is the case for c-NHEJ and high-LET induced DSBs^54,58–60^. Our CRISPR/Cas9 model system allows addressing some of these fundamental questions of genome integrity and experiments along these lines are presented next.

**Fig. 3:**
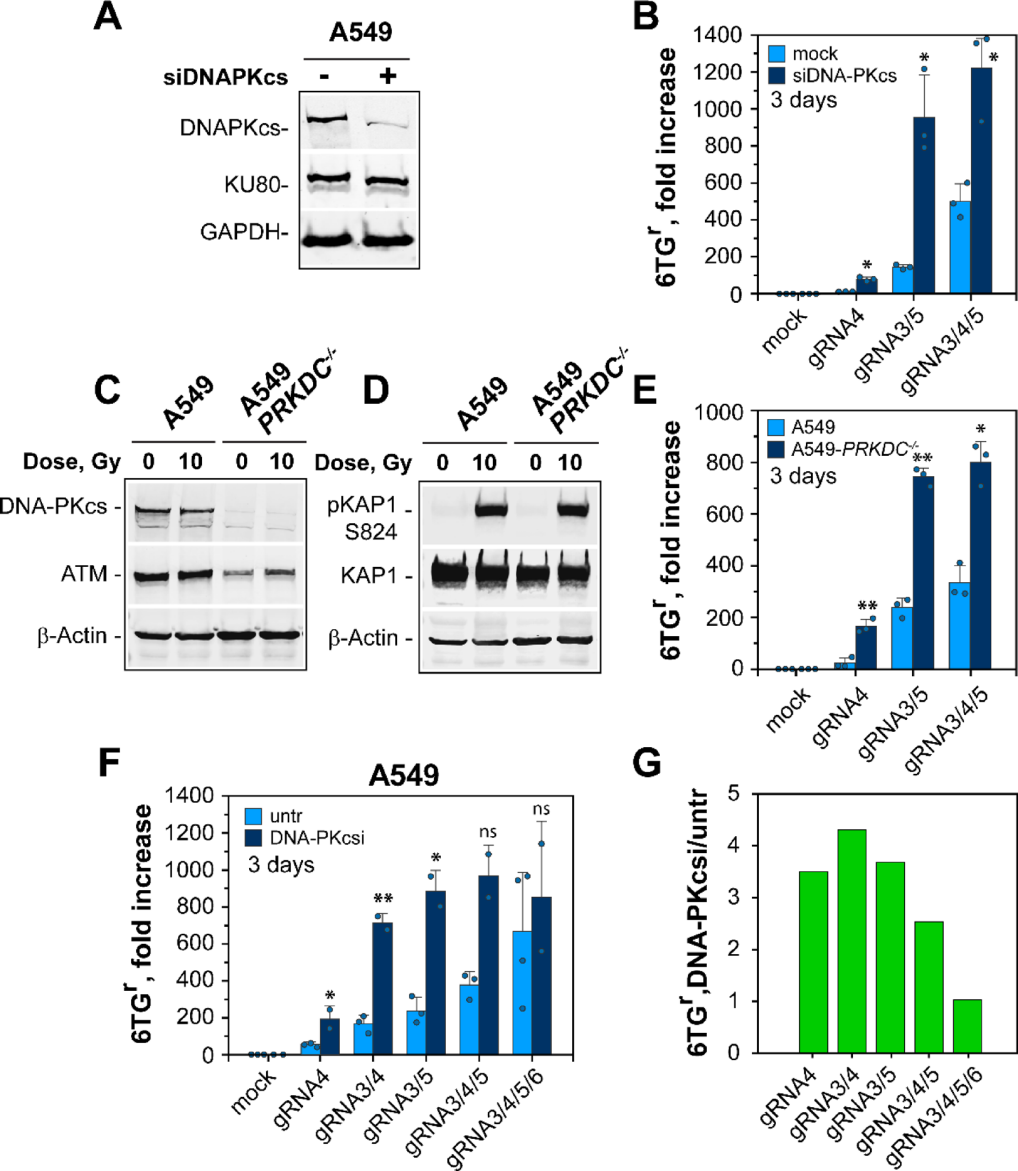
*DNA-PKcs suppresses mutation induction.* (**A**) Western blot analysis showing DNA-PKcs knockdown in A549 cells by specific siRNA. The level of KU80 and GAPDH is used as loading control. Non-cropped images are provided in Figure S4A. (**B**) Fold increase in MF of mock transfected and DNA-PKcs depleted A549 cells, 3 days after transfection with the indicated combinations of gRNA expression vectors. Data represent the mean and SD from 3 experiments. Dots indicate the raw values of each repeat. (**C**) Western blot analysis showing DNA-PKcs and ATM levels in parental A549 and A549-*PRKDC*^*−/−*^ cells. b-Actin is used as a loading control. Non-cropped western blot images are provided in Figure S4B. (**D**) Western blot analysis following phosphorylation of KAP1 at Serine-824 (pKAP1-S824), as a marker for efficient activation of the IR-induced DNA damage response. The levels of KAP1 and b-Actin are used as loading controls. Non-cropped western blot images are provided in Figure S4C. (**E**) Fold increase in MF of parental A549 and A549-*PRKDC*^*−/−*^ cells, after transfection with the indicated combinations of gRNAs. Cells are plated in 6TG-selective media 3 days after transfection. Data represent the mean and SD from 3 individual experiments. Dots represent the raw values generated for each repeat. (**F**) Fold increase in MF of untreated A549 cells and A549 cells treated with DNA-PKcs inhibitor (NU7441, DNA-PKcsi). Data represent the mean and SD from at least two experiments. Dots represent the raw values generated for each repeat. (**G**) Relative change of MF between untreated and DNA-PKcsi treated A549 cells, calculated from the data shown in Fig. 3F. P-values for all panels are shown in Table S1.

Since c-NHEJ is dominant in removing DSBs from the genome, we explored first how its inactivation affects the induction of HPRT mutations. We used siRNA to knockdown DNA-PKcs, the key component of this pathway. Western blot (WB) analysis confirmed knockdown of the protein (Fig. 3A). Notably, mutation analysis revealed a strong increase for all combinations of DSBs tested (Fig. 3B), indicating that DNA-PKcs and thus also c-NHEJ, function as a strong suppressor of *hHPRT* gene inactivation - rather than as an inducer.

Since knockdown causes only partial removal of DNA-PKcs, we extended our investigations by generating DNA-PKcs knockout mutants in A549 cells using CRISPR/Cas9 technology. WB analysis shows lack of DNA-PKcs signal in the selected knockout clone (Fig. 3C) that renders cells c-NHEJ deficient. This is additionally supported by the increased radiosensitivity of the generated DNA-PKcs deficient clone (Figure S3A). Moreover, the absence of DNA-PKcs was accompanied by a decrease in ATM levels, a response that was reported for other DNA-PKcs deficient cell lines as well^61,62^. However, the lower ATM level does not affect the activation of the ionizing radiation-induced DDR signaling, as the KAP1 phosphorylation on Serine 824 (pKAP1-S824), a specific and well-known ATM phosphorylation target, is intact in irradiated A549-*PRKDC*^*−/−*^ cells (Fig. 3D).

A549-*PRKDC*^*−/−*^ cells, as well as cells treated with DNA-PKcs inhibitor, NU7441, show a robust increase in mutation induction, regardless of DSB complexity (Fig. 3E and F), thus confirming the results using DNA-PKcs knockdown, and further supporting the role of DNA-PKcs as a strong mutation suppressor.

Notably, analysis of the relative change in MF after suppression of DNA-PKcs activity, as compared to the controls with wild-type activity, indicates that the relative effect strongly decreases with increasing DSB cluster complexity (Fig. 3G). This is in line with the concept that DSB clusters suppress c-NHEJ activity, and with our previous observations in the I-SceI-based, CHO cell system^24,33^. Evidently, when c-NHEJ is suppressed by the complexity of the DSB, inhibition of DNA-PKcs activity only has a marginal additional effect. This is similar to observations at the cell survival level, where DNA-PKcs deficient cells exposed to high-LET IR show an RBE close to one^63^.

### Alt-EJ is a key inducer of HPRT mutations

**Fig. 4:**
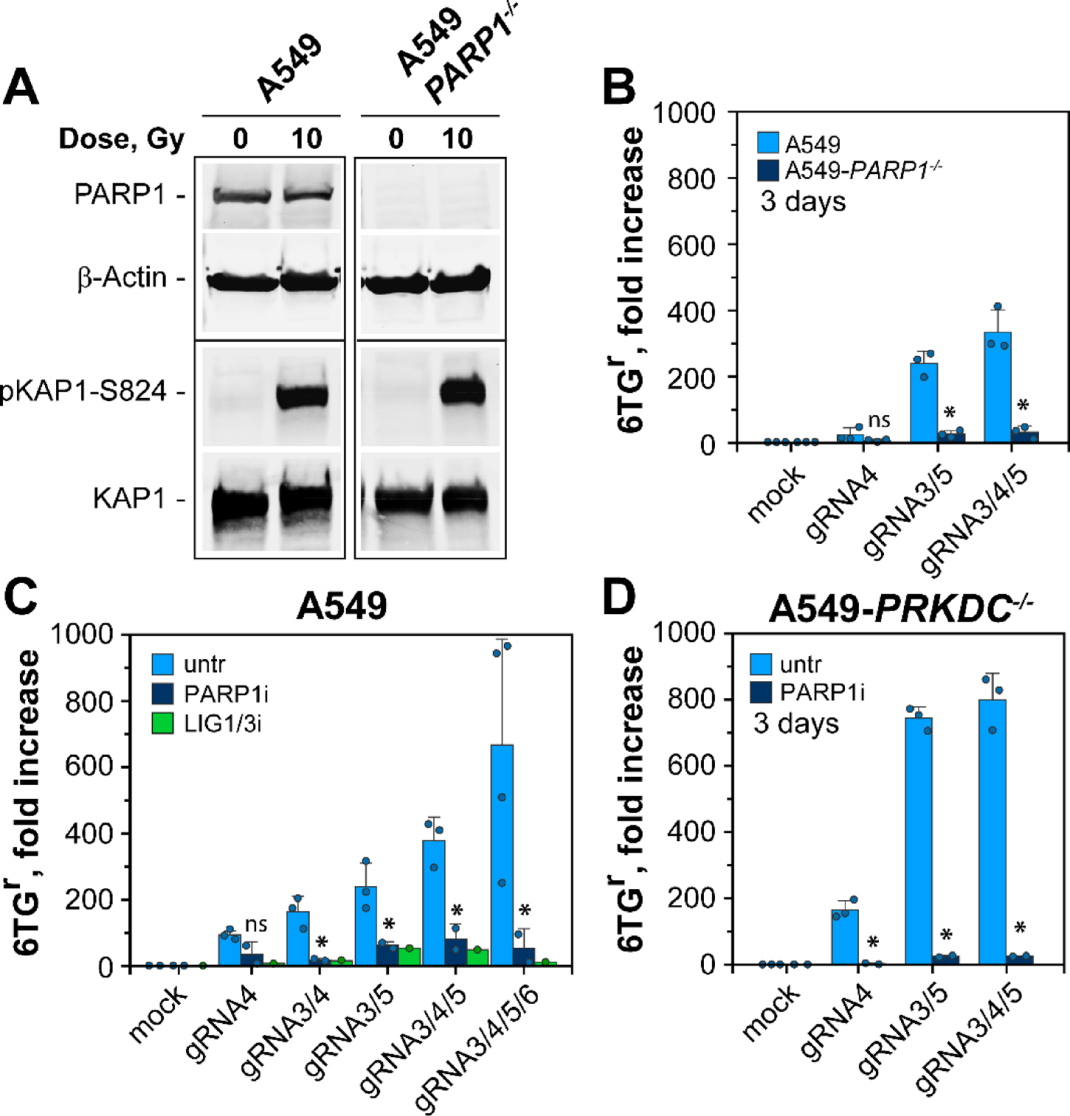
*Strong dependence of MF on alt-EJ.* (**A**) Western blot analysis showing successful knockout of PARP1 in A549-*PARP1*^*−/−*^ cells. β-Actin and KAP1 signals serve as loading controls, while pKAP1-S824 is used as marker for efficient activation of the IR-induced DDR. Non-cropped images are provided in Figures S5A and S5B. (**B**) Fold increase in MF of A549 and A549-*PARP1*^*−/−*^ cells, transfected with the indicated combinations of gRNAs. Data for the parental cell lines is replotted from Fig. 3E. Data represent the mean and SD from 3 experiments. Dots represent the raw values of each repeat. (**C**) Fold increase in MF of untreated and cells treated with PARP1 inhibitor (PJ34, PARP1i) or LIG1/3 (L67, LIG1/3i) inhibitor. Data for untreated cells is replotted from Fig. 3F. Data with PARP1 inhibitor represent the mean and SD from two biological repeats, while the data for LIG1/3 inhibition is from a single experiment. Dots represent the raw values generated in each repeat. (**D**) Fold increase of MF in A549-*PRKDC*^*−/−*^ cells, transfected with the indicated gRNAs and treated with PARP1i. Data represent the mean and SD from at least two experiments. Dots indicate the raw values generated in each repeat. P-values for all panels are shown in Table S1.

The above experiments clearly demonstrate a correlation between MF and DSB cluster complexity, but the repair pathway involved remains uncharacterized. We inquired whether alt-EJ contributes to MF, as it is frequently implicated in genomic instability. Since several studies link PARP1 activity to alt-EJ ^64–68^, we generated an A549-*PARP1*^*−/−*^ knockout cell line. Figure 4A confirms the knockout at the protein level, while Figure S3B shows loss of PARP1 activity in cells exposed to H_2_O_2_. Finally, Figure S3C shows a slight increase in radiosensitivity as a consequence of PARP1 deficiency.

Strikingly, in this mutant, HPRT mutation induction is nearly abolished (Fig. 4B). Also, a relatively specific PARP1 inhibitor, PJ34, known to inhibit alt-EJ-dependent formation of chromosomal aberrations, suppresses gene inactivation and mutation induction (Fig. 4C). Finally, since PARP1 works together with LIG3 in alt-EJ ^69–72^, we tested the effect of L67, an inhibitor of LIG1 and LIG3, with slight preference for LIG3 ^73^, on mutation induction. Notably, L67 also suppresses mutations to levels similar to those achieved with the PARP1 inhibitor (Fig. 4C).

We showed above an increase in MF following the suppression of DNA-PKcs activity. We investigated therefore, whether mutation induction in DNA-PKcs deficient cells also depends on alt-EJ. Figure 4D shows that under these conditions as well, mutation induction is fully dependent on PARP1 and was abolished following PAPR1 inhibition (Fig. 4D).

### The role of ATM in HPRT mutation induction

**Fig. 5:**
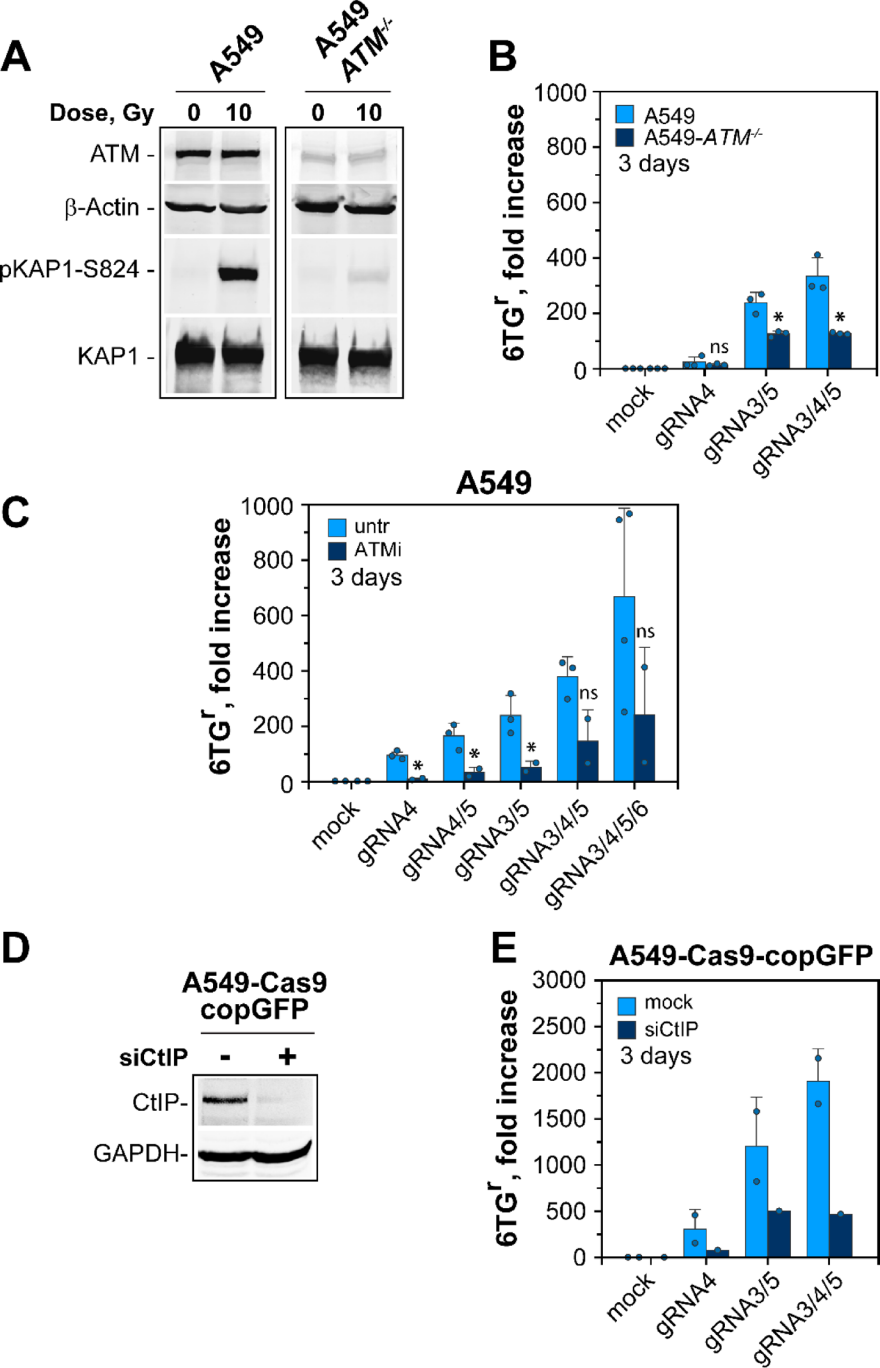
*Suppression of ATM activity and DNA end resection decrease MF.* (**A**) Western blot analysis indicating ATM knockout in A549-*ATM*^*−/−*^ cells. β-Actin and KAP1 signals serve as loading controls, while pKAP1-S824 is used as marker for efficient activation of the IR-induced DDR. Non-cropped images are provided in Figure S6A. (**B**) Fold increase of MF in parental A549 and A549-*ATM*^*−/−*^ cells, transfected with selected gRNAs. Data for parental cells is replotted from Fig. 3E. Data represent the mean and SD from 3 individual experiments. Dots represent raw values of each repeat. (**C**) Fold increase in MF in untreated A549 cells and cells treated with ATM inhibitor (KU55933, ATMi). Data for untreated cells is replotted from Fig. 3F. Data represent the mean and SD from two experiments. Dots represent the raw values of each repeat. (**D**) Western blot analysis of CtIP expression level after transfection of A549-Cas9-copGFP cells with CtIP-specific siRNA. GAPDH signal is used as a loading control. Non-cropped images are provided in Figure S6B. (**E**) Fold increase in MF of mock-transfected and CtIP-depleted A549-Cas9-copGFP cells, 3 days after transfection with the indicated combinations of gRNA expression vectors. Data for parental cell lines represent the mean and SD from two experiments, while the results for CtIP depleted cells are from a single experiment. Dots represent the raw values of each repeat. The P-values for all panels, except Fig. 5E, are shown in Table S1.

In the next set of experiments, we analyzed the effect of ATM on MF by generating a knockout cell line. WB analysis shows barely detectable levels of ATM protein in the selected A549-*ATM*^*−/−*^ clone (Fig. 5A) and functional assays fail to detect ATM activity (Figs. 5A and S3D). Specifically, phosphorylation of KAP1 at Serine 824 (pKAP1-S824), was almost undetectable in A549-*ATM*^*−/−*^ cells (Fig. 5A). Moreover, indirect IF experiments confirmed the loss of ATM activity, as no phosphorylation of ATM at Serine 1981 (pATM-S1981), an autophosphorylation marker site for ATM activation, was detected in irradiated A549-*ATM*^*−/−*^ cells (Figure S3D). Moreover, the A549-*ATM*^*−/−*^ cells were radiosensitive when exposed to x-rays (Figure S3E).

Transfection of A549-*ATM*^*−/−*^ cells with Cas9 and combinations of gRNA expression vectors, indicate that the potential of DSB clusters to generate mutations at the human *HPRT* gene is decreased (Fig. 5B). These results are also confirmed by observations in A549 cells treated with the ATM inhibitor, KU55933. In this experiment, ATM inhibition also decreases the potential of single DSBs and DSB clusters to induce mutations (Fig. 5C).

Data showing the involvement of ATM in DNA end resection have been reported^74–76^. Moreover, other experiments demonstrate that ATM deficient cells experience an activation delay in DNA end resection^77^. This is in line with the results shown here.

DNA end resection is a key process that facilitates the engagement of alt-EJ, by revealing microhomologies^78–80^. We therefore tested the role of DNA end resection itself in mutation induction by knocking down its key regulator, CtIP (Fig. 5D). In these experiments we tested A549 cells constitutively expressing Cas9, together with a fluorescent protein marker (copGFP) that allows control of expression levels. Figure 5E shows that in this cell line, single DSBs and DSB clusters generated by transfection solely with the appropriate gRNAs induce mutations more efficiently than in the biological systems utilized in the experiments discussed above that also require the transfection with the Cas9 expression vector. This is likely due to the continuous expression of Cas9 in the cells that increases cutting efficiency. Notably, in CtIP-depleted cells, generation of single DSBs or DSB clusters strongly suppresses MF (Fig. 5E). However, suppression of resection has smaller effects than inhibition of PARP1 or LIG1/3 activities, suggesting that mutational events persist at conditions of reduced resection. This aspect requires further investigations. We conclude that this cell line has distinct advantages for the line of investigation we are pursuing and plan to use it extensively in future work testing in detail not only the endpoints described above, but also more fundamental questions of the role of DSB complexity in gene inactivation and mutation induction.

## Discussion

The diversity and spectrum of mechanisms involved in the repair of DSBs underscores the cell’s intolerance for unrepaired DSBs and justifies the evolution of repair pathways with reduced demands on the break, but prone of generating genomic instability. Previous studies on IR-induced mutations at the HPRT locus reported genomic alterations mainly in the form of large deletions, deriving from normal or complex DSBs, as classically defined^35–40^.

Because classically defined complex DSBs have not being modelled in vivo as of yet, while the alternative forms of complexity, e.g., DSB clusters, can be modelled in vivo using genomic engineering approaches, we focused our recent work on this topic. Indeed, it has been shown that two or more closely spaced DSBs along the DNA can be induced by IR, and are the likely culprits of the increased efficacy of high-LET radiation^10,24,33,34^.

In the present study we developed a system based on CRISPR/Cas9 technology that allows to target specific sequences in the *hHPRT* gene and induce site-specific DSBs and DSB clusters of increasing complexity, to study their biological consequences. Our results show that DSB clusters of four DSBs, spaced within a 600 bp region at distances of 150–300 bp, have the highest propensity of generating mutations (Fig. 1A and D), suggesting that DSB cluster complexity is a major factor driving errors in DNA rejoining leading to mutations. Our results also demonstrate that DSBs must be spaced within a maximum distance not exceeding ~ 600 bp to synergize in mutation induction. This finding is consistent with earlier studies suggesting that the proximity of DSBs can influence repair pathway choice, with closely localized DSBs more likely to engage error-prone repair mechanisms^24,33^. However, it is important to note that DSBs separated by ~ 100 bp fail to show this synergism^33^ and that distance phenomena are likely to be strongly dependent on local chromatin organization at the sites where single DSBs or DSB clusters are induced. We anticipate that further development of the CRISPR/Cas9 approach to mutation induction may allow to investigate biological responses of DSBs or DSB clusters generated in distinct chromatin compartments, e.g., eu- vs. heterochromatin, replicative vs. non-replicative, or transcribed vs. non-transcribed chromatin regions.

While the CRISPR/Cas9 technology allows induction of single DSBs and DSB clusters at selected genomic locations to investigate the effects of their processing on the genome, mimicking high-LET IR-induced effects at other endpoints can be much more demanding. For example, to biologically model the effects of Cas9-generated DSBs or DSB clusters on cell survival, multiple DSBs (or DSB clusters) will be required at different locations in the genome – not as straightforward task as targeting a single locus with easy integrity assessment methods, as is the case with the *HPRT* gene. Furthermore, the type of DSBs that Cas9 generates differs from the type induced by IR ^10,52^. Clearly, further developments will be required for the realistic approximation of IR-induced DSBs and their complexities. We envision further developments of our system, allowing the induction of DSB clusters of different complexity at multiple strategically selected genomic locations to investigate large scale chromosomal aberrations and possibly identify “hot spots” in the genome for structural chromosomal abnormalities (SCAs).

The observations reported above advance our understanding of the mutagenic potential of DSB clusters and highlight the underpinning repair mechanisms. We documented that DSB clusters, induce mutations more effectively than single DSBs and that the mutation frequency increases by increasing the number of DSBs within the cluster. This observation confirms and extends our previous research showing that DSB clusters induce more efficiently SCAs, that could increase cell lethality^24,33,34^.

Moreover, our findings also suggest that DSB clusters suppress c-NHEJ and shift the overall DSB repair processing to highly mutagenic DSB repair mechanisms (Fig. 3B, E and F). This is in line with our previous results on the topic^24,33^. Our current study extends and consolidates this observation and shows that DNA-PKcs deficiency results in increased MF, irrespective of DSB cluster complexity (Fig. 3). Interestingly, however, the contribution of DNA-PKcs and thus of c-NHEJ to the repair of DSB clusters decreases with increasing DSB cluster complexity, which is in line with our postulate that c-NHEJ is inherently inhibited at complex DSB clusters induced by high-LET IR.

Our work also characterizes repair pathways that are engaged in the repair of DSB clusters. We show that the increased mutagenicity of clustered DSBs is primarily mediated by the DNA end resection-dependent alt-EJ (Fig. 4), which can function as backup when c-NHEJ is compromised. Moreover, we characterized LIG3 and possibly LIG1 as factors involved in the mechanism underpinning mutation induction from DSB clusters (Fig. 4C). These findings align well with previous studies implicating alt-EJ in the formation of chromosomal aberrations, particularly in contexts where c-NHEJ was inhibited^81–84^.

Experiments with ATM-deficient cells, or administration of ATM inhibitors, show marked reduction in mutation induction (Fig. 5B and C). We postulate that this reflects the requirement for ATM activity in the regulation of DDR in general and DNA end resection in particular, as well as in facilitating DSB repair through HR and alt-EJ pathways^74,85–88^. Interestingly, ATM deficiency plays only a limited role in mutation induction when DSB clusters of 3 or 4 DSBs are induced. It is likely that when more complex DSB clusters are generated, DNA end resection may be promoted by alternative mechanisms, possibly involving the activity of ATR. Indeed, our results on the regulation of G_2_-checkpoint after exposure to high-LET radiation demonstrate a strong dependence of ATR^77^. The role of DNA end resection in mutation induction is also confirmed by downregulating the CtIP, an essential DNA end resection protein (Fig. 5E). However, the incomplete suppression of HPRT mutation under these conditions may partly reflect the generation of “blunt” DSB ends by Cas9, that could suppress DNA end resection initiation. Moreover, although c-NHEJ efficiency is reduced at DSB clusters, some c-NHEJ activities could still bind to open DSB ends, thus blocking their further processing.

In summary, our work provides valuable insights into the mechanisms of DSB repair when DSB clusters are generated. By demonstrating the enhanced mutagenic potential of DSB clusters and elucidating the roles of various repair pathways, this study contributes to our understanding of the biological consequences of high-LET IR modalities and the potential of DSB clusters as complex DSBs. Detailed sequencing analysis of the *hHPRT* locus in mutants generated under the conditions employed here, which we plan in the near future, will further enhance our conclusions and will extend the mechanistic insights of shifts in DSB repair pathways balance as a function of DSB complexity.

## Supplementary Information

Below is the link to the electronic supplementary material.

Supplementary Material 1

## Data Availability

All data is available upon request to the corresponding authors.
