# Supplementary material for: CRISPR/Cas9 generated DSB clusters mimic complex lesions induced by high-LET radiation and shift repair from c-NHEJ to mutagenic repair pathways
Source: Sci Rep. 2025 Oct 20;15:36480. doi: 10.1038/s41598-025-22945-9 (PMC12537819; doi:10.1038/s41598-025-22945-9)
Supplement: Supplementary file 1 — Supplementary Material 1 [file 41598_2025_22945_MOESM1_ESM.pdf]

SUPPLEMENTARY INFORMATION

CRISPR/Cas9 generated DSB-clusters mimic complex lesions induced by high-LET radiation and shift repair from c-NHEJ to mutagenic repair pathways

Emil Mladenov, Mathias Kallies, Martin Stuschke, Eleni Gkika, and George Iliakis

Supplementary Figure Legends

Figure S1: (A) Schematic representation of Exon 3 of the *hHPRT* gene showing the target sites of the utilized guide RNAs and the primers (in green) that were used to amplify the selected region for the T7E1 assay. The distances between DSBs and the primers are indicated in base pairs (bp). The distances between the primer pairs reflect the size of the PCR products that is amplified in the T7E1 assay. **(B)** Representative agarose gel electrophoresis images showing the PCR products amplified from genomic DNA (gDNA) templates isolated from cells co-transfected with Cas9 and the indicated gRNAs expressing vectors. PCR products were generated by amplification of genomic DNA using the indicated primer pairs for the individual gRNAs. The PCR products were incubated with T7E1 that cleaves the DNA heteroduplexes generated as a result of error-prone gRNA-mediated DSB processing. Representative agarose gels from selected individual experiments are shown. **(C)** Quantitative densitometry analysis of cleaved products released after T7E1 incubation. The percentage of cleaved product is calculated from densitometry analysis of four technical repeats and is plotted as bars, representing the mean and standard deviation (SD). The individual dots represent the values determined in each individual repeat. **(D)** Representative immunofluorescence images of 53BP1 foci in Cas9-GFP positive cells after transfection of A549 cells with selected gRNA vectors. The same pull of cells is used for gDNA isolation utilized in T7E1 analysis. Two sets of images are shown. **(E)** Representative immunofluorescence images of 53BP1 foci in Cas9-GFP positive cells after transfection with vectors expressing the indicated single gRNAs. **(F)** Representative immunofluorescence images of γH2AX foci in Cas9-GFP positive cells after transfection with the indicated single gRNA expressing vectors.

Figure S2:(A) Representative flow cytometry dot plots of A549 cells mock-transfected or transfected with a GFP expressing plasmid to assess transfection efficiency (TE). This control was carried out in every experiment of the project and the results were used to correct MFs. The upper panels show the gating of viable cells, while the lower panels show gating for GFP positive (GFP+) cells in the viable cell population. **(B)** Representative colonies of A549 cells treated with increasing concentrations of 6TG, indicated in black and the corresponding plating efficiency (PE) shown in red. **(C)** Mutation frequency of HPRT in A549 cells transfected with the indicated gRNAs and plated for colony formation in 6TG-supplemented media at the indicated time intervals (2, 3, or 4 days). As a pilot experiment, the results represent a single repeat. **(D)** Representative 60 mm dishes showing colonies of 6TG-resistant A549 cells,

transfected with the indicated combinations of gRNA expression vectors. The calculated, not normalized, MFs are shown in red.

Figure S3: **(A)** Colony formation analysis of parental (wt) and DNA-PKcs deficient (A549-*PRKDC*$^{-/-}$) cells, irradiated with increasing x-rays doses. **(B)** Representative IF images of parental (A549) and PARP1-deficient (A549-*PARP1*$^{-/-}$) cells, treated with H_2O_2 and stained with anti-PAR-specific antibody to visualize PARP1 activity. **(C)** Colony formation analysis of parental (wt) and A549-*PARP1*$^{-/-}$ cells irradiated with increasing doses of x-rays. **(D)** Representative IF images of parental and ATM-deficient A549 cells stained with pATM-S1981 antibody to detect the active form of ATM after irradiation with 1 Gy of x-rays. **(E)** Colony formation analysis of parental (wt) and ATM-deficient (A549-*ATM*$^{-/-}$) cells, irradiated with increasing radiation doses. All results represent the mean values from at least two biological repeats, while the error bars reflect the SD.

Figure S4: **(A)** Raw, uncropped, western blot images of the results presented in Figure 3A. Exp1 and Exp2 annotation indicates the samples generated in separate repeats. **(B)** Raw, uncropped, western blot images of the results presented in Figure 3C. **(C)** Raw, uncropped, western blot images of the results presented in Figure 3D. For all panels, the molecular weight of the protein marker lanes is on the left, while the name of the detected protein is shown on the right.

Figure S5: **(A)** Raw, uncropped western blot images of the results presented in Figure 4A, upper part. **(B)** Raw, uncropped western blot images of the results presented in Figure 4A, lower part. The Molecular weight of the protein marker lane is indicated on the left, while the name of the detected protein on the right.

Figure S6: **(A)** Raw, uncropped, western blot images of the results shown in Figure 5A. **(B)** RAW, uncropped, western blot images of the results shown in Figure 5D. The molecular weight of the protein marker bands is indicated on the left, while the name of the detected protein on the right. The non-relevant lanes are indicated as NA; Exp1 and Exp2 identify samples generated in separate experiments.

Table S1: P-values calculated for the results plotted in the graphs from the indicated figures. The following annotations were used to indicate significance levels: ns ($P > 0.05$), * ($P \leq 0.05$), ** ($P \leq 0.01$), *** ($P \leq 0.001$).

Table S2: Raw values of mutation frequency from four selected experiments used to calculate the fold increase of MF showed in Figures 1D and 2A.

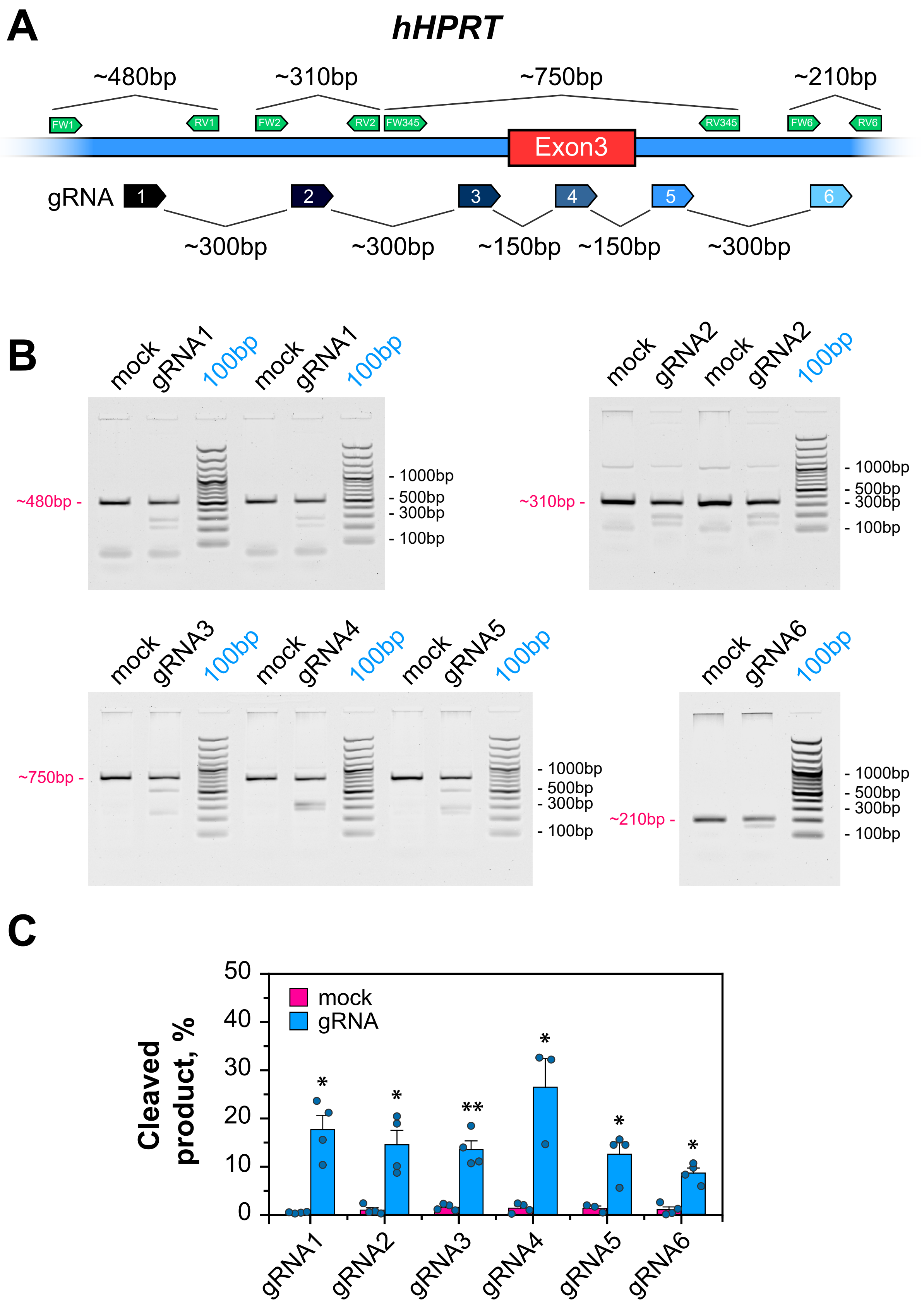

Mladenov et al. **Figure S1**

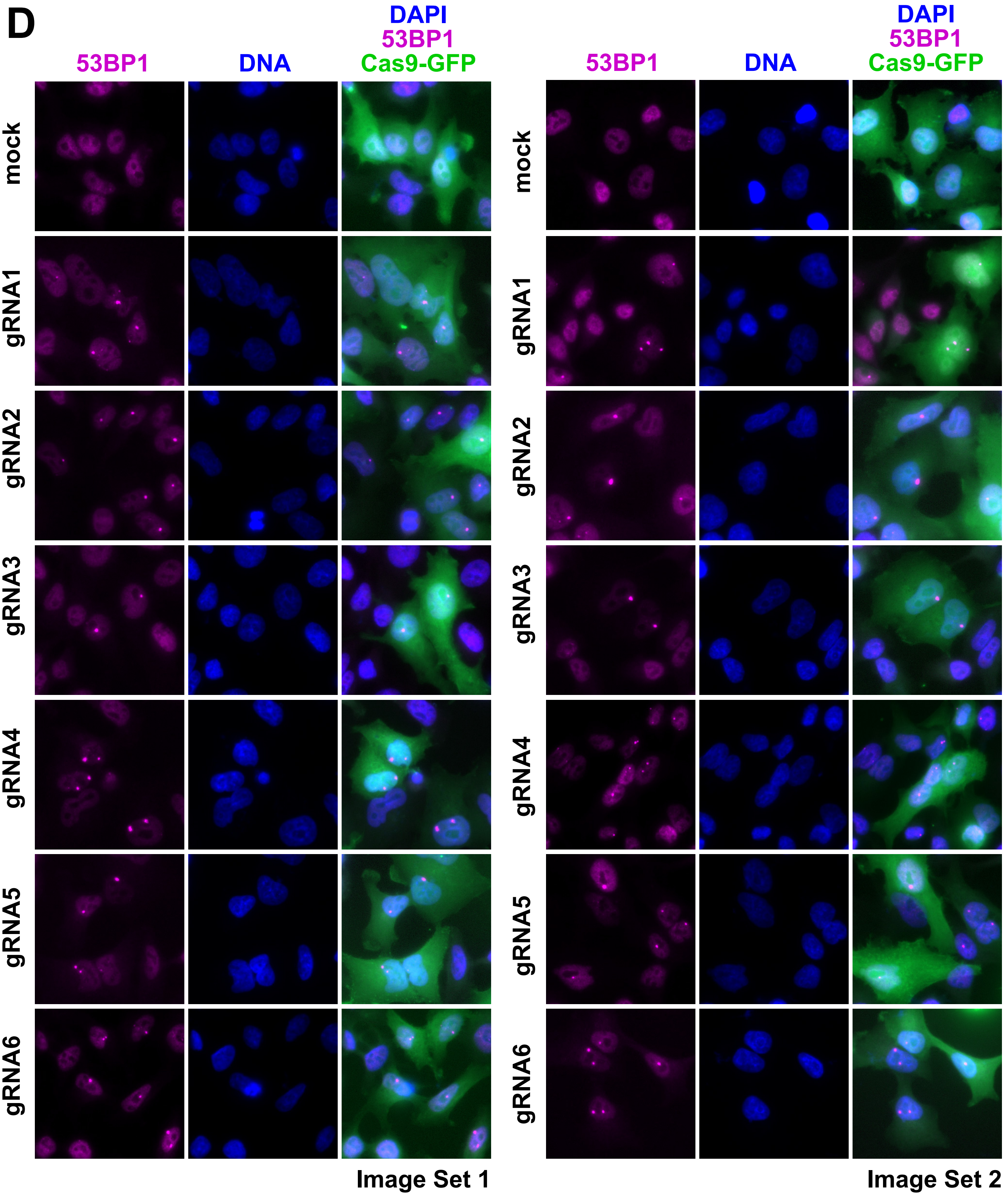

Mladenov et al. **Figure S1**

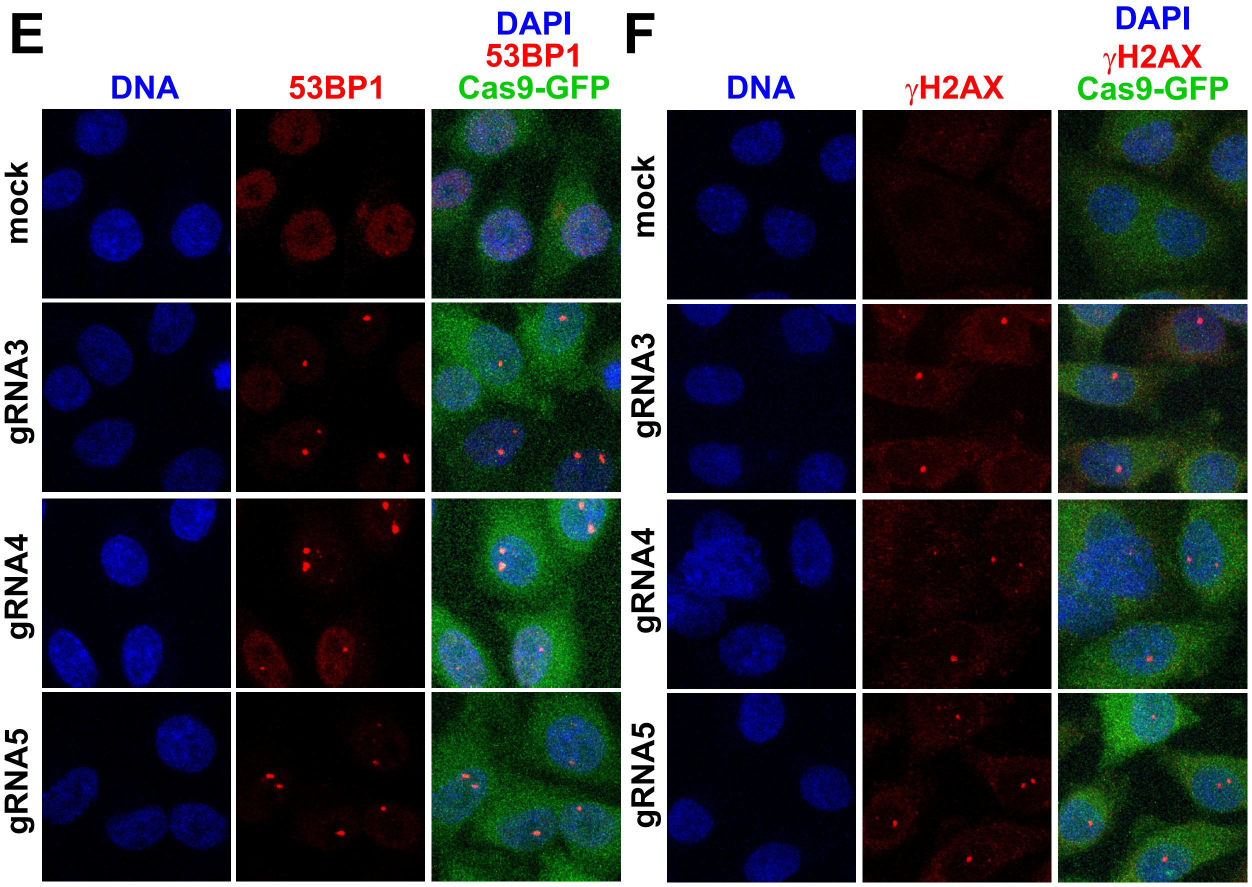

Mladenov et al. **Figure S1**

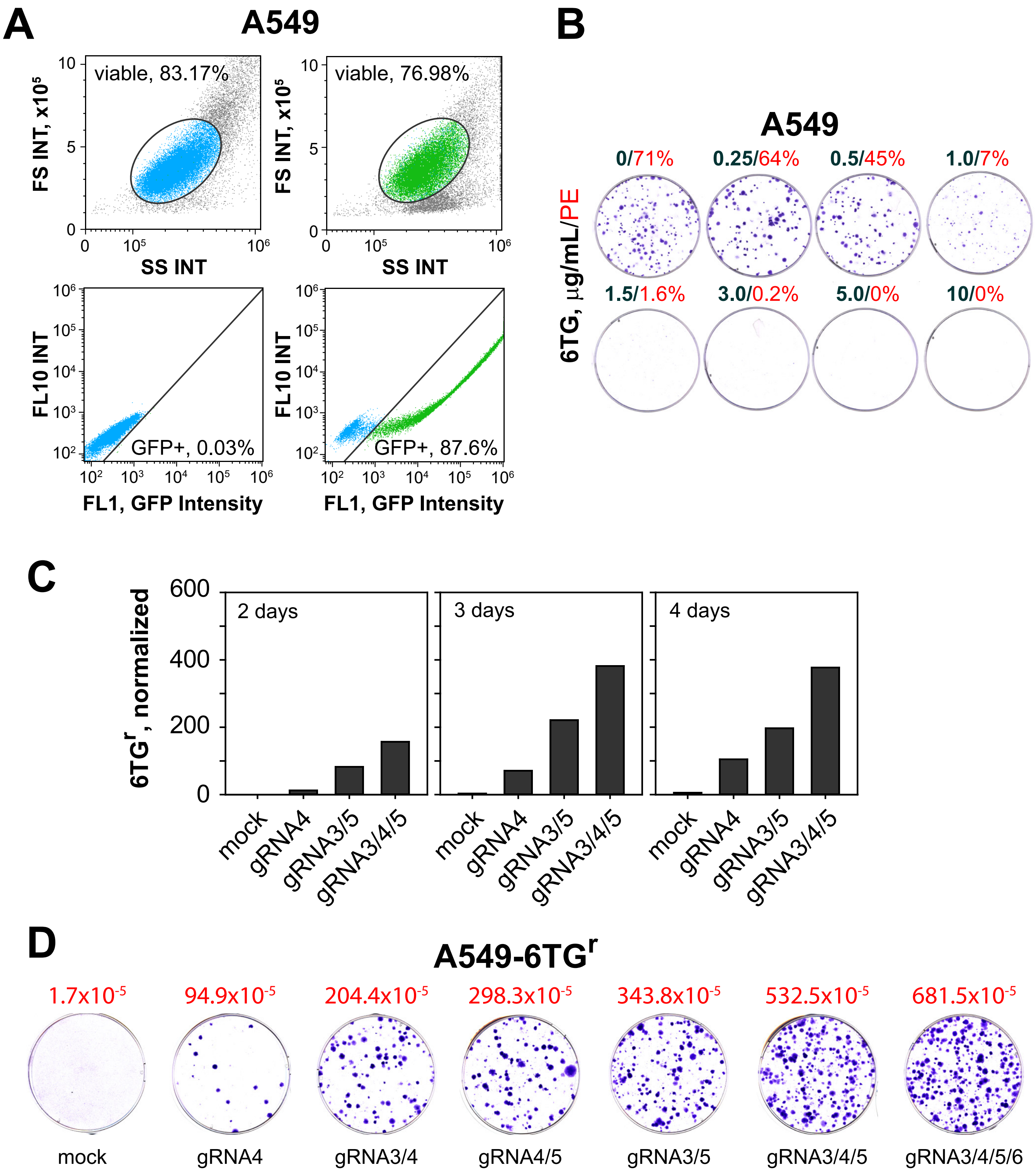

Mladenov et al. **Figure S2**

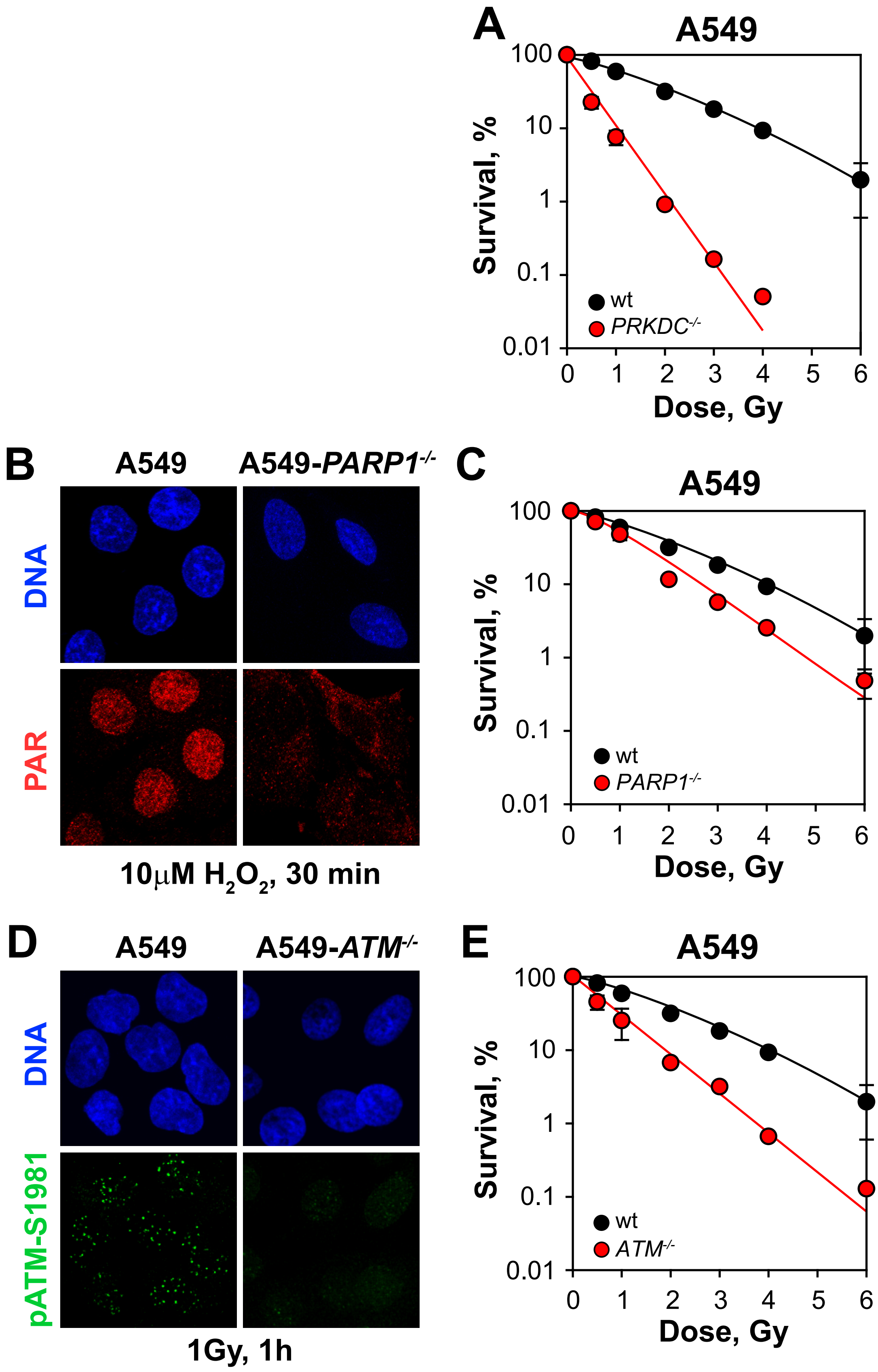

Mladenov et al. **Figure S3**

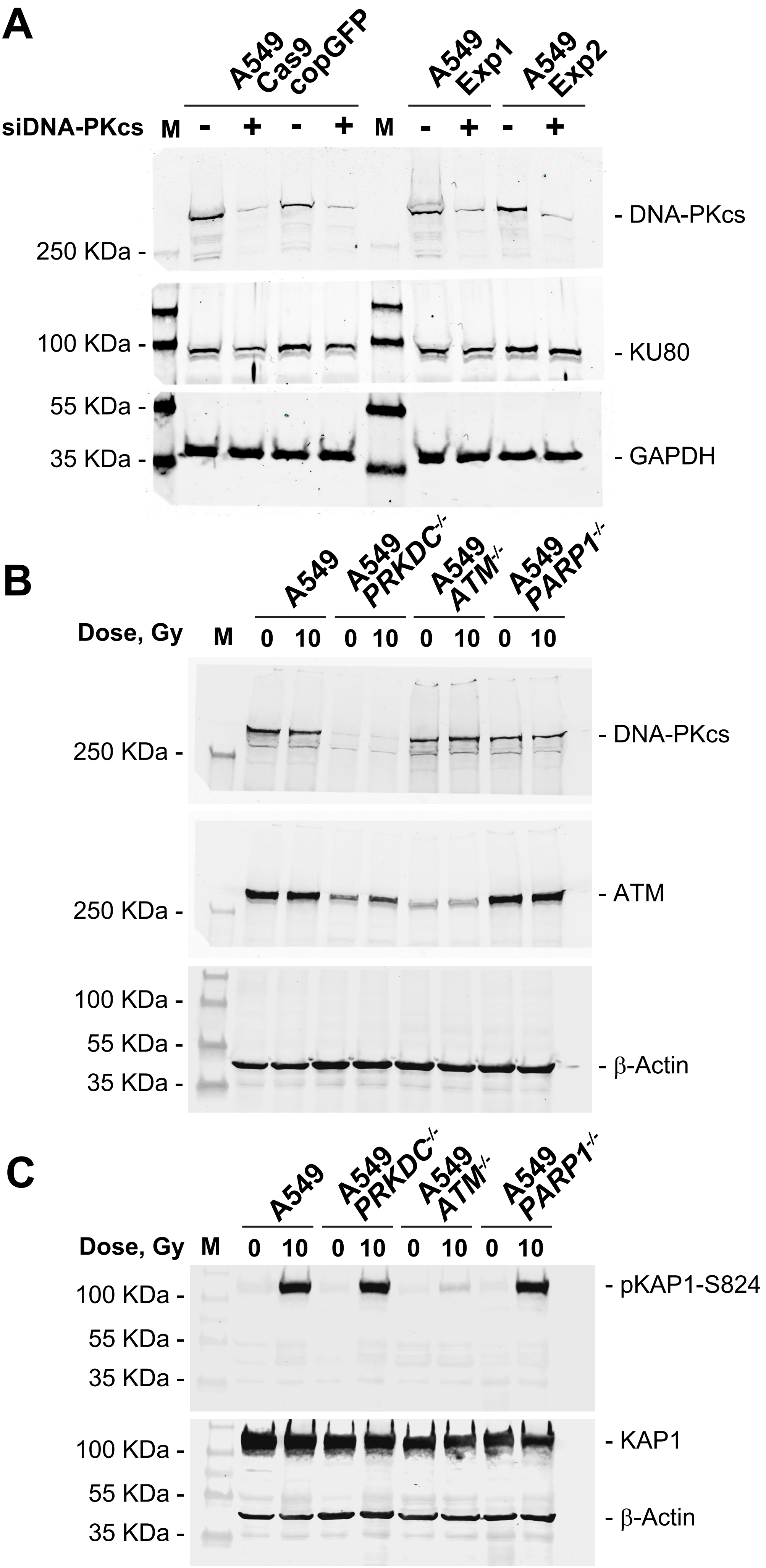

Mladenov et al. Figure S4

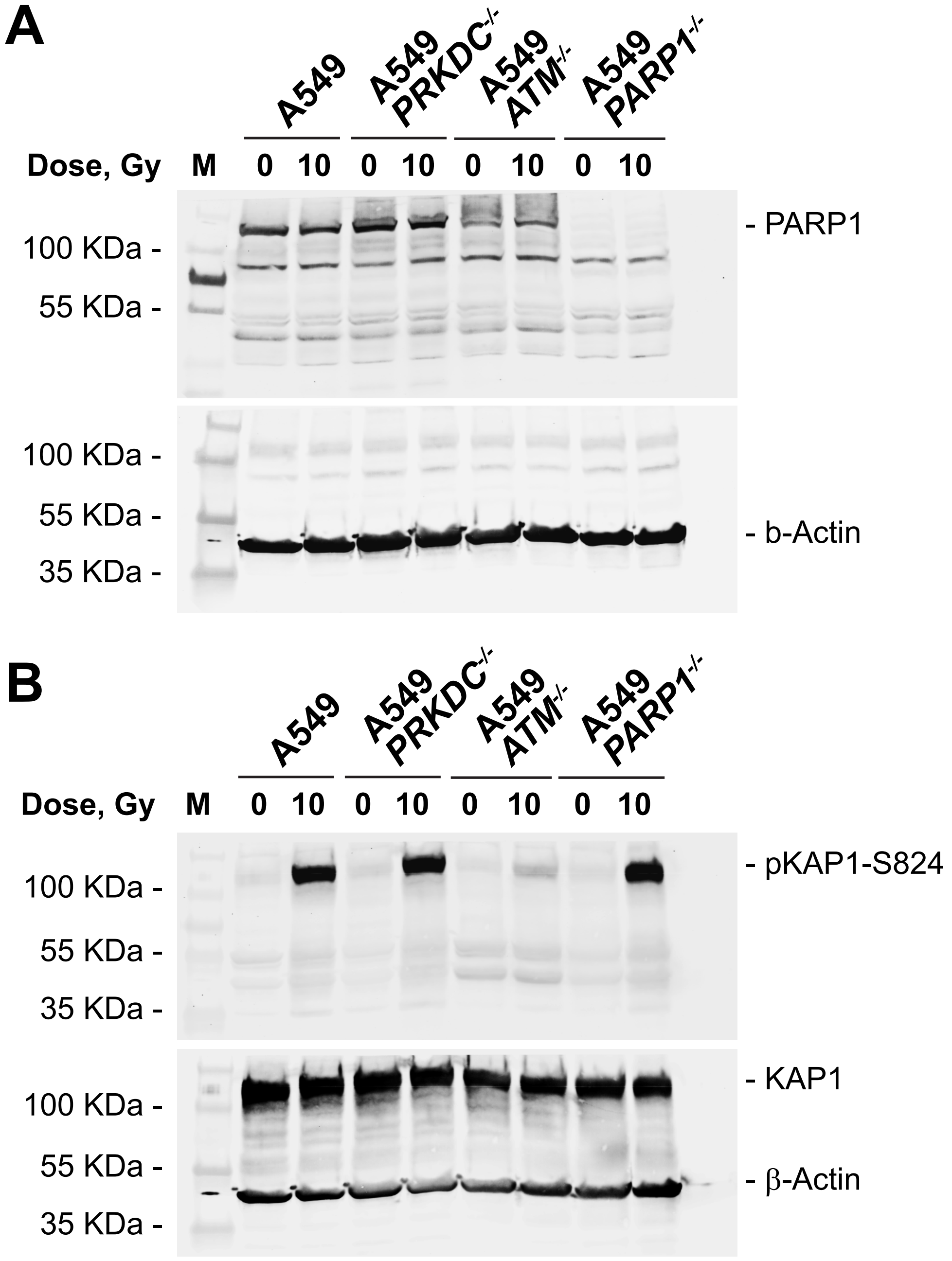

Mladenov et al. **Figure S5**

A

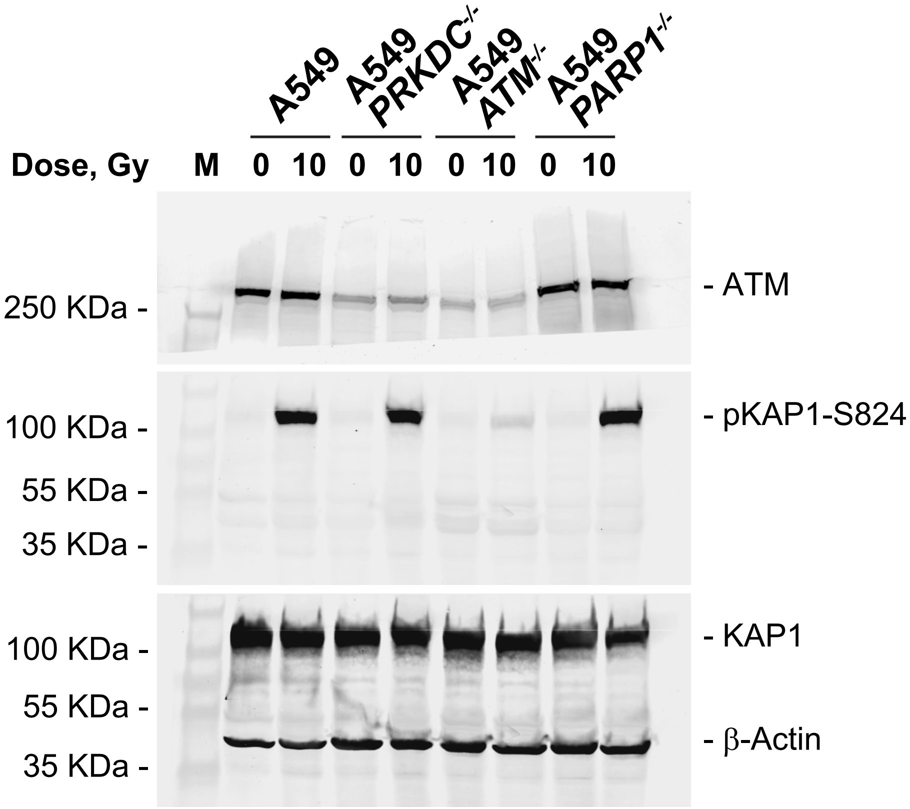

B

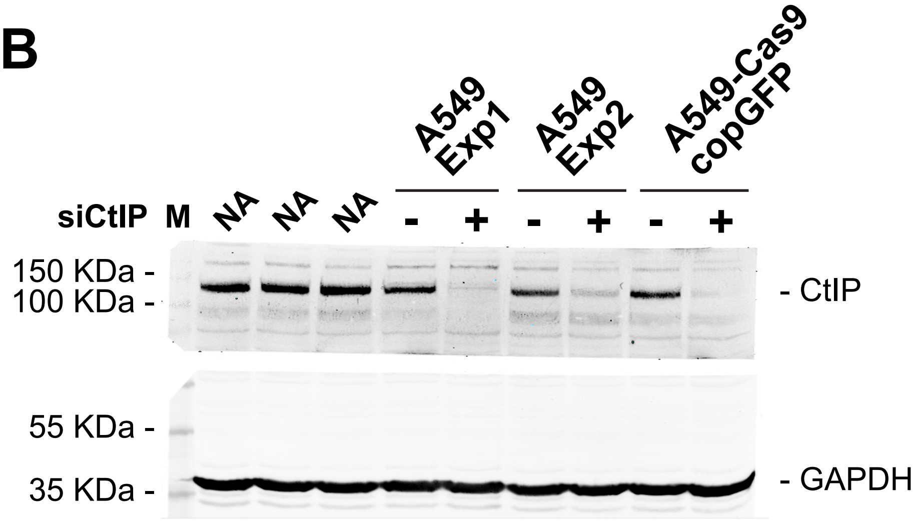

Mladenov et al. **Figure S6**

Figure 1C

P-Values	gRNA1	gRNA2	gRNA3	gRNA4	gRNA5	gRNA6
mock	0.7494	0.2247	0.2445	0.0105	0.1858	0.8297
Significance	ns	ns	ns	*	ns	ns

Figure 1D

P-Values	gRNA4	gRNA3/4	gRNA4/5	gRNA3/5	gRNA3/4/5	gRNA3/4/5/6
mock	0.3623					
gRNA4		0.0376	0.0160	0.0099	0.0034	<0.0001
Significance	ns	*	*	**	**	***

Figure 2A

P-Values	gRNA4	gRNA3/4	gRNA4/5	gRNA3/5	gRNA3/4/5	gRNA3/4/5/6
mock	0.2822					
gRNA4		0.0146	0.0074	<0.0001	<0.0001	<0.0001
Significance	ns	*	**	***	***	***

Figure 2B

P-Values	gRNA4	gRNA3/4	gRNA4/5	gRNA3/5	gRNA3/4/5	gRNA3/4/5/6
mock	0.2822					
gRNA4		0.0095	<0.0001	0.0163	0.7186	0.0033
Significance	ns	**	***	*	ns	**

Figure 2C

P-Values	gRNA4	gRNA3/4	gRNA4/5	gRNA3/5	gRNA2/6	gRNA1/6
mock	0.0309					
gRNA4		0.9603	0.0178	<0.0001	0.7186	0.4297
Significance	*	ns	*	***	ns	ns

Figure 3B

P-Values	gRNA4	gRNA3/5	gRNA3/4/5
mock/siRNA	0.0112	0.0236	0.0218
Significance	*	*	*

Figure 3E

P-Values	gRNA4	gRNA3/5	gRNA3/4/5
wt/PRKDC$^{-/-}$	0.0014	0.0051	0.0315
Significance	**	**	*

Figure 3F

P-Values	gRNA4	gRNA3/4	gRNA3/5	gRNA3/4/5	gRNA3/4/5/6
Untr/Tr	0.0501	0.0060	0.0205	0.1748	0.3182
Significance	*	**	*	ns	ns

Figure 4B

P-Values	gRNA4	gRNA3/5	gRNA3/4/5
wt/PARP1$^{-/-}$	0.2166	0.0113	0.0254
Significance	ns	*	*

Figure 4C

P-Values	gRNA4	gRNA3/4	gRNA3/5	gRNA3/4/5	gRNA3/4/5/6
Untr/Tr	0.4911	0.0290	0.0499	0.0151	0.0314
Significance	ns	*	*	*	*

Figure 4D

P-Values	gRNA4	gRNA3/5	gRNA3/4/5
Untr/Tr	0.0288	0.0101	0.0133
Significance	*	*	*

Figure 5B

P-Values	gRNA4	gRNA3/5	gRNA3/4/5
wt/ATM$^{-/-}$	0.4927	0.0183	0.0358
Significance	ns	*	*

Figure 5C

P-Values	gRNA4	gRNA3/4	gRNA3/5	gRNA3/4/5	gRNA3/4/5/6
Untr/Tr	0.0483	0.0517	0.0502	0.1105	0.1415
Significance	*	*	*	ns	ns

ns P > 0.05, * P ≤ 0.05, ** P ≤ 0.01, *** P ≤ 0.001

Mladenov et al. **Table S1**

Figure 1D

Raw Data	mock	gRNA4	gRNA3/4	gRNA4/5	gRNA3/5	gRNA3/4/5	gRNA3/4/5/6
Exp1	1.05×10^{-5}	148.08×10^{-5}	214.9×10^{-5}	472.86×10^{-5}	333.35×10^{-5}	450.42×10^{-5}	992.96×10^{-5}
Exp2	1.48×10^{-5}	121.11×10^{-5}	167.73×10^{-5}	258.56×10^{-5}	NA	442.1×10^{-5}	757.71×10^{-5}
Exp3	1.7×10^{-5}	94.9×10^{-5}	204.4×10^{-5}	298.3×10^{-5}	343.8×10^{-5}	532.5×10^{-5}	681.5×10^{-5}
Exp4	1.86×10^{-5}	165.71×10^{-5}	413.56×10^{-5}	205.88×10^{-5}	416.83×10^{-5}	502.57×10^{-5}	1591.61×10^{-5}

Figure 2A

Raw Data	mock	gRNA4	gRNA3/4	gRNA4/5	gRNA3/5	gRNA3/4/5	gRNA3/4/5/6
Exp1	1.24×10^{-5}	39.28×10^{-5}	64.68×10^{-5}	91.84×10^{-5}	244.4×10^{-5}	215.4×10^{-5}	257.38×10^{-5}
Exp2	1.31×10^{-5}	85.36×10^{-5}	212.82×10^{-5}	157.81×10^{-5}	394.14×10^{-5}	360.82×10^{-5}	416.55×10^{-5}
Exp3	1.25×10^{-5}	17.26×10^{-5}	29.16×10^{-5}	101.96×10^{-5}	100.95×10^{-5}	150.05×10^{-5}	NA
Exp4	0.25×10^{-5}	7.08×10^{-5}	20.54×10^{-5}	26.53×10^{-5}	40.71×10^{-5}	47.3×10^{-5}	123.2×10^{-5}

Mladenov et al. **Table S2**
